# Synaptic encoding of temporal contiguity

**DOI:** 10.3389/fncom.2013.00032

**Published:** 2013-04-12

**Authors:** Srdjan Ostojic, Stefano Fusi

**Affiliations:** ^1^Department of Neuroscience, Center for Theoretical Neuroscience, Columbia University Medical CenterNew York, NY, USA; ^2^Department Etudes Cognitives, CNRS, Group for Neural Theory, LNC INSERM U960, Ecole Normale SuperieureParis, France

**Keywords:** synaptic plasticity, learning and memory, temporal contiguity, Markov processes, forgetting

## Abstract

Often we need to perform tasks in an environment that changes stochastically. In these situations it is important to learn the statistics of sequences of events in order to predict the future and the outcome of our actions. The statistical description of many of these sequences can be reduced to the set of probabilities that a particular event follows another event (temporal contiguity). Under these conditions, it is important to encode and store in our memory these transition probabilities. Here we show that for a large class of synaptic plasticity models, the distribution of synaptic strengths encodes transitions probabilities. Specifically, when the synaptic dynamics depend on pairs of contiguous events and the synapses can remember multiple instances of the transitions, then the average synaptic weights are a monotonic function of the transition probabilities. The synaptic weights converge to the distribution encoding the probabilities also when the correlations between consecutive synaptic modifications are considered. We studied how this distribution depends on the number of synaptic states for a specific model of a multi-state synapse with hard bounds. In the case of bistable synapses, the average synaptic weights are a smooth function of the transition probabilities and the accuracy of the encoding depends on the learning rate. As the number of synaptic states increases, the average synaptic weights become a step function of the transition probabilities. We finally show that the information stored in the synaptic weights can be read out by a simple rate-based neural network. Our study shows that synapses encode transition probabilities under general assumptions and this indicates that temporal contiguity is likely to be encoded and harnessed in almost every neural circuit in the brain.

## 1. Introduction

When we see clouds in the morning, we carry an umbrella with us. The life of any animal depends on its faculty to predict the future based on the current situation and previous experiences. The prediction of the future is often based on our memory of how often a particular event follows a sequence of other events (Bar, 2011). In many situations we can predict the immediate future by using associations between events that have been repeatedly temporally contiguous or separated by short time intervals, as for the conditioned and the unconditioned stimulus in classical conditioning protocols.

Temporal contiguity plays an important role in various learning paradigms and for this reason, it has been widely studied. Studies on classical conditioning investigated the formation of behavioral associations between sensory stimuli and their dependence on temporal contingencies (Rescorla, 1988). Studies on human (Kahana, 1996) and primate (Miyashita, 1988; Sakai and Miyashita, 1991; Yakovlev et al., 1998) memory revealed that subjects naturally remember the temporal order of events, even when this information is behaviorally irrelevant (Cleeremans and McClelland, 1991). Studies on visual processing suggest that view-invariant representations of objects are achieved by merging the neural representations of different views, which are temporally contiguous when we manipulate the objects (Wallis et al., 1993; Li and DiCarlo, 2008, 2010).

The mechanisms proposed for reproducing the rich phenomenology of the experiments or for modeling the cognitive functions based on temporal contiguity range from abstract algorithms, designed to explain the observed behavior, to detailed models of biological neural networks. For example, experiments on classical conditioning are usually modeled in the theoretical framework of the Rescorla–Wagner theory (Rescorla and Wagner, 1972). The generation of short (as in pair associates tasks) and long deterministic temporal sequences is often achieved by asymmetric synaptic couplings, which express the covariance of patterns of activity that are temporally contiguous in the sequence (Sompolinsky and Kanter, 1986). These models essentially work as hetero-associative memories as each recognized pattern of activity representing an item in a sequence leads to the retrieval of the pattern of activity that represents the next item. This idea has been implemented in several models that focused on the role of hippocampus in the consolidation of sequence memory (Minai and Levy, 1993; Jensen and Lisman, 1996; Levy, 1996; Wallenstein and Hasselmo, 1998; Wallenstein et al., 1998) and on a few variations of serial list learning (Lewandowsky and Murdock, 1989). The same idea has been used in attractor neural networks models that can represent associations between temporally contiguous sensory stimuli in the spatial distributions of the patterns of neural activity that are elicited by individual stimuli (Griniasty et al., 1993; Amit et al., 1994; Brunel, 1994, 1996; Mongillo et al., 2003). These attractor models have been tested against experiments (Yakovlev et al., 1998) and have been recently extended to the formation of a hierarchy of temporal context representations (Rigotti et al., 2010a). The process of formation of view invariant representations has been modeled as a process of extraction of slowly co-occurring visual features (Wiskott and Sejnowski, 2002).

**Figure 1:**
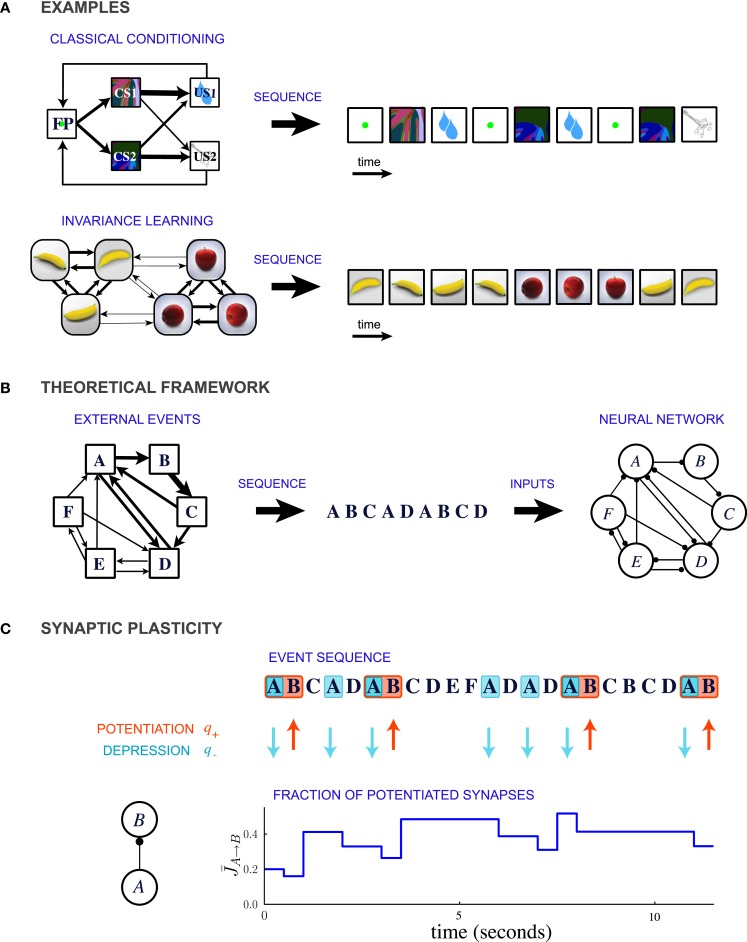
**Description of the model. (A)** Two examples of learning paradigms. Top: A probabilistic trace-conditioning task similar to the one studied in Paton et al., 2006 (FP, fixation point; CS1, CS2, conditioned stimuli; US1, US2, unconditioned stimuli). Bottom: Schematic illustration of natural viewing of three-dimensional objects leading to invariance learning (Wallis et al., 1993; Li and DiCarlo, 2008, 2010). In both examples, the temporal statistics of events are represented in the diagram on the left, where the widths of the arrows represent the transition probabilities between the different events. At each time-step an event is generated stochastically according to these transition probabilities, giving rise to a temporal sequence (right). **(B)** General theoretical framework. Events occur sequentially in a probabilistic order. The order of the events is specified by the matrix of transition probabilities, indicated schematically by the widths of the connections in the leftmost diagram. Each event activates a population of neurons, and the sequence of events leads to plastic modifications of the synapses between the populations. **(C)** Illustration of synaptic dynamics for synapses between two neural populations encoding events *A* and *B*. The synaptic dynamics are determined by the sequence of external events: potentiation occurs when the post-synaptic event B takes place after the pre-synaptic event A; depression occurs when the pre-synaptic event takes place and the post-synaptic population is inactive (PRE-activated depression rule, see text and Figure 2 for other depression rules). Each potentiating (resp. depressing) event modifies a fraction *q*_+_ (resp. *q*_−_) of bistable synapses projecting from population *A* to population *B* (in the illustration, *q*_+_ = 0.3 and *q*_−_ = 0.2).

In this theoretical study, we propose that a common, unifying mechanism could underlie the encoding of temporal contiguity in a variety of learning paradigms. We represent the learning paradigms implicating temporal contiguity as special cases of a general conceptual framework in which events occur in probabilistic sequences (Figure 1B). For example, classical conditioning corresponds to the situation where the order of the occurrence of events is highly asymmetric (Figure 1A top). In contrast, learning invariant representations of three-dimensional objects involves events which are organized in clusters that represent different objects (Figure 1A bottom).

We examine the dynamics of model synapses connecting neural populations that encode distinct events. We show that these synapses naturally encode the temporal statistics of events provided that the synaptic modifications depend on sequences of consecutive events. These findings extend previous studies on synaptic encoding of the probability of individual events (Rosenthal et al., 2001; Soltani and Wang, 2006; Fusi et al., 2007) to the more general case of arbitrarily long sequences of events that occur stochastically.

## 2. Methods

### 2.1. External events and neural encoding

We consider an environment in which events occur stochastically. We assume that there is a finite set of events {*E*_*i*_}_*i* = 1,…, *n*_. As we are interested only in the temporal order of events and not in their actual timing, we assume that time is discrete and in every time step one event occurs. The transition probabilities *P*_*E*_*j*__ → *E*_*i*_ determine the probability of observing event *E*_*i*_ at time step *t* + 1 if event *E*_*j*_ was observed at time *t*. We assume that the event sequence is Markovian, so that the matrix of transition probabilities *T*_*ij*_ = *P*_*E*_*j*__ → *E*_*i*_ fully specifies the statistics of the sequences. The frequencies at which individual events occur are given by the eigenvector of *T* associated with a unit eigenvalue.

The sequence of external events drives the dynamics of a neural network. In the network, every event is encoded by a distinct population of neurons: when event *E*_*i*_ occurs, the corresponding neural population *i* is activated. We assume that different neural populations do not overlap, so that every neuron is activated by only one external event. In general, cortical recordings show the neurons are selective to more than one event (Yamane et al., 2006). Although we will consider only the case in which the neural representations of different events do not overlap, our analysis can easily be extended to the random sparse representations, (i.e., when overlap between neural populations is small), and, more in general, to the synapses connecting the non-overlapping parts of distributed dense representations.

We also assume that during learning the neural activity is imposed by a sufficiently strong external input, and it does not depend on the state of the plastic synaptic connections that we are studying.

The Markov transition matrices used in the simulations were generated randomly using the following procedure:

1. The number of external events was selected (*n* = 12 in the displayed results).
2. For each event, the number of events that could follow with non-zero transition probabilities was chosen randomly between 2 and 4 (the number of events that could follow was limited in order to avoid having only small transition probabilities).
3. These events were chosen randomly among the set of all *n* events, and transition probabilities were chosen randomly between 0 and 1 with a uniform distribution and then they were normalized.

Other ensembles of transition matrices have also been explored, the reported results are independent of the precise ensemble.

### 2.2. Synaptic plasticity rules

We assume that the synapses possess a finite number *m* of states that are stably preserved on long timescales. These states are numbered, so that each state corresponds to an integer *k*, with 1 ≤ *k* ≤ *m*. With the exception of Figure 6, we focus on bistable synapses (*m* = 2), which have only a depressed (*k* = 1) and a potentiated (*k* = 2) state.

The occurrence of an event induces transitions between different synaptic states that are governed by the plasticity rules. At time *t*, potentiation of synapses occurs if one of the two following conditions are satisfied: (1) the pre- and post-synaptic neurons are both activated at the same time *t* and (2) the pre-synaptic neuron is activated at time *t* − 1 and the post-synaptic neuron is activated at time *t*. The first type of potentiation affects only the synapses connecting neurons belonging to the same population. In this study, we assume that the individual events are familiar to the network, so that the synapses connecting neurons belonging to the same population have reached equilibrium and their strength is constant. We therefore study only the dynamics of synapses connecting distinct neural populations, for which only the second type of potentiation plays a role. When the condition for potentiation is fulfilled, if the synapse is not in the maximally potentiated state (*k* < *m*), the synaptic state is increased by a unit with probability *q*_+_.

For depression we considered three different possibilities:

- *PRE-activated depression*: the synapse is depressed when the pre-synaptic population is activated at time *t* but the post-synaptic population is not active at that same time step.
- *POST-activated depression*: the synapse is depressed when the post-synaptic population is activated at time *t* but the pre-synaptic population is not active at that same time step.
- *Unspecific depression*: the synapse is depressed at every time step, independently of the event that occurs.

In each case, when the conditions for depression occur, if the synapse is not in the maximally depressed state (*k* > 1), the synaptic state is depressed by a unit with probability *q*_−_.

The synaptic strength *J* of a population of equivalent synapses is defined as

(1) $$J=\frac{1}{m-1}\sum_{k\;=\;1}^{m}\left(k-1\right)\rho_{k},$$

where ρ_*k*_ is the fraction of synapses in the *k*th state. For bistable synapses, *J* is simply the fraction of synapses in the potentiated state.

Our mathematical analysis, developed in full detail in the section Appendix A, is valid for a much wider class of plasticity rules than described here. The results are derived for bistable synapses, in the framework of a mean-field theory. The dynamics are first analyzed for arbitrary potentiation and depression probabilities *q*_+_ and *q*_−_. In a second step, we analyze the limit of slow learning in which the probabilities *q*_+_ and *q*_−_ are small, so that a relatively large number of events are needed to potentiate or depress an individual synapse. In this limit, the mean synaptic strength and the transient timescale afford simple expressions that are easy to interpret and moreover turn out to be identical to the results obtained by neglecting correlations in the synaptic dynamics.

The results obtained in the slow learning limit appear to be quantitatively accurate also in the case of relatively large potentiation and depression probabilities (i.e., *q*_+_ and *q*_−_ larger than 0.1).

### 2.3. The dynamics of populations of bistable synapses

As each neural population consists of a large number of neurons, two populations are connected by a large number of statistically equivalent synapses. We describe the dynamics of the bistable synapses projecting from neural population *j* to neural population *i* by tracking the fraction *J*_*j* → *i*_ of potentiated synapses. If the number of bistable synapses connecting the neural populations is large, the dynamics of the fraction *J*_*j* → *i*_ are given by

(2) $$\begin{array}{l} J_{j\;\to \;i}(t+1)=J_{j\;\to \;i}(t)+q_{+}(1-J_{j\;\to \;i}(t))\xi_{j\;\to \;i}^{+}(t,t-1)\\ \;-\;q_{-}J_{j\;\to \;i}(t)\xi_{j\;\to \;i}^{-}(t) \end{array}$$

where ξ^+^_*j* → *i*_(*t*, *t* − 1) is a function which is one if the external events at time *t* − 1 and *t* are such that potentiation occurs, and zero otherwise. For example, for the potentiation rule described above, ξ^+^_*j* → *i*_(*t*, *t* − 1) = 1 if *E*_*j*_ occurred at time *t* − 1 and *E*_*i*_ occurred at time *t*. Similarly ξ^−^_*j* → *i*_(*t*) = 1 if the event at time *t* is such that depression occurs, and zero otherwise.

In the simulations of synaptic dynamics, we directly use Equation (2), i.e., we assume that the synaptic populations consist of large numbers of synapses. Equivalently this can be seen as tracking the average state of a single bistable synapse over many instantiations of the synaptic transition probabilities, for a fixed sequence of external events. This second interpretation is used for the mathematical analysis presented below.

### 2.4. Averaged dynamics of bistable synapses

In previous studies, which focused on uncorrelated sequences of external events, the outcome of synaptic dynamics was studied by averaging Equation (2) over sequences of external events (Brunel et al., 1998; Soltani and Wang, 2006; Fusi et al., 2007). This approach, however, cannot be directly extended to our situation for two reasons: (1) the sequences of external events are correlated in time and (2) the synaptic potentiation at time step *t* depends on the sequence of events that occurred at two previous consecutive time steps. More precisely, averaging Equation (2) over all possible sequences of events (we denote this average by angle brackets) involves computing 〈*J*_*j* → *i*_(*t*)ξ^+^(*t*, *t* − 1)〉 and 〈*J*_*j* → *i*_(*t*)ξ^−^(*t*)〉. As the value *J*_*j* → *i*_(*t*) depends on the external event that occurred at time step *t* − 1, and both ξ^+^(*t*, *t* − 1) and ξ^−^(*t*) are correlated with that event, the averages 〈*J*_*j* → *i*_(*t*)ξ^+^(*t*, *t* − 1)〉 and 〈*J*_*j* → *i*_(*t*)ξ^−^(*t*)〉 cannot be factorized in contrast to the previously studied case where successive events were uncorrelated, and potentiation depended only on the current time step.

Our detailed mathematical analysis (see Appendix) nevertheless shows that, when the learning rates *q*_+_ and *q*_−_ are small, the correlations between *J*_*j* → *i*_(*t*) and ξ^+^(*t*, *t* − 1) as well as ξ^−^(*t*) can be neglected, so that the synaptic dynamics averaged over external sequences are well approximated by

(3) $$\langle \delta J_{j\;\to \;i}\rangle (t)=q_{+}\left(1-\langle J_{j\;\to \;i}\rangle (t)\right)f_{j\;\to \;i}^{+}-q_{-}\langle J_{j\;\to \;i}\rangle (t)f_{j\;\to \;i}^{-}$$

where δ*J*_*j* → *i*_(*t*) = *J*_*j* → *i*_(*t* + 1) − *J*_*j* → *i*_(*t*) is the synaptic increment, and *f*^+^_*j* → *i*_ = 〈ξ^+^_*j* → *i*_〉 and *f*^−^_*j* → *i*_ = 〈ξ^−^_*j* → *i*_〉 are the frequencies of occurrence of potentiating and depressing events (or sequences of events).

The steady state synaptic strength ${\bar{J}}_{j\to i}$ can be obtained from Equation (3) by setting the synaptic increment 〈δ*J*_*j* → *i*_〉 to zero, which directly leads to Equation (15). The transient timescale τ_*j* → *i*_ given in Equation (17) corresponds to the inverse of the factor multiplying 〈*J*_*j* → *i*_〉 in Equation (3).

The fact that the correlations can be neglected in the slow learning limit is proved rigorously, but it is possible also to provide an intuitive argument. When the learning rates are small, the chances that a synapse is updated in two consecutive time steps are small. As a consequence, even if the two potential synaptic modifications are highly correlated, the synapses will not be affected by these correlations, as they will rarely be modified in two consecutive trials. Most of the time they will not be modified at all, and only with small probability they will be modified once. More in general, if the largest learning rate is *q*_*L*_, whenever 1/*q*_*L*_, the average time between two synaptic modifications, is substantially larger than the autocorrelation time of the Markov process, the correlations can be neglected. Notice that the transition probabilities between two consecutive events can still be encoded even when the correlations are neglected. Indeed, they are encoded in individual synaptic modifications (which depend on two consecutive events), and they are not affected by any mechanism that makes negligible the correlations between two consecutive synaptic modifications (which would depend on three events).

### 2.5. Equilibrium state for synapses with *m* > 2 states

For synapses with an arbitrary number *m* of states the equilibrium synaptic strength is given by

(4) $${\bar{J}}_{j\;\to \;i}=F(f_{j\;\to \;i}^{+}/f_{j\;\to \;i}^{-})$$

where *f*^+^_*j* → *i*_ and *f*^−^_*j* → *i*_ are the frequencies of occurrence of potentiating and depressing events (or sequences of events) and the transfer function *F* is given by

(5) $$F(x)=\frac{1}{m-1}\left(\frac{\frac{q_{+}}{q_{-}}x}{1-\frac{q_{+}}{q_{-}}x}+\frac{m{\left(\frac{q_{+}}{q_{-}}x\right)}^{m}}{{\left(\frac{q_{+}}{q_{-}}x\right)}^{m}-1}\right).$$

For synapses with a large number of states *m*, the transfer function *F* becomes a sigmoid centered at *q*_−_/*q*_+_. The derivation of this transfer function is given in Appendix B.

### 2.6. Effective model of fluctuations in the synaptic dynamics

Using the mathematical expression for the transient timescale given in Equation (17), we constructed an effective model of synaptic dynamics in which an effective synaptic weight at time *t* was computed directly from the sequence of events that occurred until time *t*. More precisely, the effective synaptic weight at time *t* is given by *F*(*N*^+^_*ij*_/*N*^−^_*ij*_) where *N*^+^_*ij*_ and *N*^−^_*ij*_ are the numbers of potentiating and depressing events that occurred between times *t* − τ_*j* → *i*_ and *t*, and *F* is the transfer function between probabilities and synaptic weights given in Equation (14).

The standard deviation of the synaptic weights around the steady-state value can be easily computed for the effective model and reads

(6) $$\Delta J_{j\;\to \;i}^{2}={\left(F^{\prime }(P_{E_{j}\;\to \;E_{i}})\right)}^{2}\frac{P_{E_{j}\;\to \;E_{i}}(1-P_{E_{j}\;\to \;E_{i}})}{2f_{j}\tau_{j\;\to \;i}}.$$

### 2.7. Neural dynamics

Our study of synaptic dynamics in response to sequences of external events makes no assumptions on the details of neural dynamics. However, to illustrate how synaptic plasticity impacts the neural dynamics, we adopted a particular model of neural dynamics.

For the sake of simplicity, we model the dynamics of the network on a population level, using a firing-rate model which is essentially a simplified version of a biologically realistic network of spiking neurons (Brunel and Wang, 2001; Wang, 2002; Wong and Wang, 2006). This firing-rate model exhibits a phenomenology similar to the biologically realistic network while allowing for a more direct analysis.

The network consists of *n* populations of excitatory neurons and a single population of inhibitory neurons, *n* being the number of distinct external events that can occur. The activity of the *i*th excitatory population is described by the dynamics of its firing rate *r*_*i*_, given by

(7) $$\tau_{m}\frac{d}{dt}r_{i}=-r_{i}+\Phi (I_{\text{syn}}^{\left(i\right)})+\sigma \eta_{i}(t).$$

Here τ_*m*_ is the time constant of firing rate dynamics (τ_*m*_ = 10 ms in the simulations), *I*^(*i*)^_syn_ is the total synaptic input to population *i*, described below, η_*i*_ is a white noise process uncorrelated between neural populations, σ is the noise amplitude (σ = 0 unless otherwise specified) and Φ is a threshold-linear transfer function defined by

$$\Phi (x)=\{\begin{array}{cc} gx & \text{if }x>\text{0}\\ 0 & \text{if }x<\text{0} \end{array}$$

where *g* is the gain of the population (*g* = 450 in the simulations).

The dynamics of the inhibitory population are assumed to be instantaneous and directly proportional to the excitatory activity in the network (Brunel and Wang, 2001; Wong and Wang, 2006). The firing rate *r*_*I*_ is therefore given by

(8) $$r_{I}=gI_{\text{syn}}^{\left(I\right)}$$

where *I*^(*I*)^_syn_ is the total synaptic input to the inhibitory population, specified below.

In the biologically realistic network (Brunel and Wang, 2001; Wang, 2002; Wong and Wang, 2006), it has been shown that NMDA-based synapses play a central role, while AMPA-based synapses are not crucial. We therefore include only NMDA synapses in our network. The dynamics of the average gating variable *S*_*j*_ of synapses projecting from the population *j* to other neural populations are given by

(9) $$\tau_{s}\frac{d}{dt}S_{j}=-S_{j}+g_{s}r_{j}$$

where τ_*s*_ is the time constant of NMDA synapses, and *g*_*s*_ is a scaling constant.

The total input current *I*^(*i*)^_syn_ received by the excitatory population *i* is given by

(10) $$I_{\text{syn}}^{\left(i\right)}=\sum_{j}J_{j\;\to \;i}S_{j}-J_{I}r_{I}+h_{i}(t)$$

where *J*_*j* → *i*_ is the strength of synapses projecting from neural population *j* to neural population *i*, *h*_*i*_(*t*) is an external input activated when event *E*_*i*_ takes place, and *J*_*I*_ represents the strength of the inhibitory synapses. For *i* ≠ *j*, the synaptic strength *J*_*j* → *i*_ was determined during learning, i.e., *J*_*j* → *i*_ = *F*(*P*_*E*_*j*__ → *E*_*i*_), where *F* is the synaptic transfer function defined in Equation (14). We also include non-vanishing recurrent synaptic strengths *J*_*i* → *i*_ which is identical for all populations.

The total input current *I*^(*I*)^_syn_ to the inhibitory population is given by

(11) $$I_{\text{syn}}^{\left(I\right)}=\sum_{j}J_{E\;\to \;I}S_{j}$$

where *J*_*E* → *I*_ is the strength of the synapses that excitatory neurons make on inhibitory neurons.

As the transfer function of the inhibitory population is linear (Equation 8), replacing Equation (11) the input current to excitatory neurons becomes

(12) $$I_{\text{syn}}^{\left(i\right)}=\sum_{j}\left(J_{j\;\to \;i}-g_{I})S_{j}+h_{i}(t\right)$$

where *g*_*I*_ = *gJ*_*I*_*J*_*E* → *I*_ is the overall strength of the inhibition, which determines the effective interactions between the excitatory neurons. The inhibition strength was systematically varied in simulations.

For Figure 7, the transition probabilities between external events are *P*_*A* → *B*_ = 0.65, *P*_*A* → *C*_ = 0.35, *P*_*B* → *D*_ = 0.9, *P*_*B* → *E*_ = 0.1, *P*_*C* → *D*_ = 0.55, and *P*_*C* → *E*_ = 0.55. The ratio between potentiation and depression is *q*_+_/*q*_−_ = 1. The neural time constant is τ_*m*_ = 10 ms, and the NMDA time constant is τ_*s*_ = 100 ms. The external input is constant pulse of amplitude *h* = 0.05 and duration 100 ms. The recurrent synaptic strength is *J*_*i* → *i*_ = 0.02 for all *i*. In the three panels, the inhibition strengths *g*_*I*_ are (1) *g*_*I*_ = 0.5, (2) *g*_*I*_ = 0.4, and (3) *g*_*I*_ = 0.3. The value of the gain is *g* = 450. Note that for simplicity the values of all inputs was normalized to vary between 0 and 1, and the scales are set by the gain constants *g* = 450 and *g*_*s*_ = 0.02.

## 3. Results

Our aim is to study how the temporal statistics of external events are encoded in the distribution of synaptic efficacies. We consider a situation in which the temporal sequences of events are generated by a Markov process (i.e., each event occurs stochastically with a probability that depends only on the previous event). The events occur stochastically at a fixed temporal interval, the length of which could range from tens of milliseconds to seconds. The transition probability from one event to another expresses quantitatively how often the two events are temporally contiguous. The set of the transition probabilities between all possible pairs of events (transition matrix) fully characterizes the statistics of the temporal sequences. Such a theoretical framework encompasses a number of experimental paradigms in which temporal contiguity has been studied (see Figure 1A for examples).

The occurrence of each external event induces the activation of a specific pattern of neural activity (Figure 1B). For simplicity we assume that every neuron responds to one event only, so that every event activates a distinct population of neurons. Moreover, we assume that during learning the neuronal dynamics are dominated by the inputs representing the external event, so that the interactions within the network are negligible. The temporal sequence of neuronal activations induced by the sequence of events causes long term synaptic modifications and determines the distribution of synaptic weights. We will show that, when the synaptic dynamics relaxes to equilibrium, the distribution of the synaptic weights connecting neurons that respond to two different events encodes the temporal contiguity between these two events.

The synaptic distribution will in general depend on the synaptic dynamics and in particular on how the long term modifications are induced by the sequence of pre and post-synaptic neural activity. To analyze this dependence, we considered synapses that have only a finite number of stable states that can be preserved on long time scales. This allows us to greatly simplify the description of the synaptic dynamics because a wide class of detailed synaptic models can be reduced to the set of transition probabilities between stable states following the occurrence of a relevant event that is encoded by the activity of the pre and post-synaptic neurons. All short term processes that do not lead to a consolidated (long term) synaptic modification (i.e., a transition to a different state) can be ignored without any loss of generality, as long as they do not affect the next long term synaptic modification. For examples of how detailed biophysical models can be reduced to this description see Mongillo et al., 2003 and Drew and Abbott (2006).

In most of this study we focus on synapses with two states (depressed and potentiated), but we also examine the impact of the number of states on our results. Bistable synapses always relax to an equilibrium distribution in a stationary environment and they are compatible with recent experimental results (Petersen et al., 1998; O'Connor et al., 2005), although other experiments show that there could be more than two stable states (Enoki et al., 2009). Moreover, bistability is also compatible with detailed and more abstract biophysical models of synaptic dynamics (Crick, 1984; Zhabotinsky, 2000; Graupner and Brunel, 2007). Finally, bistable synapses seem to be representative of a wide class of realistic synaptic models when the memory performance is considered (Fusi and Abbott, 2007).

**Figure 2:**
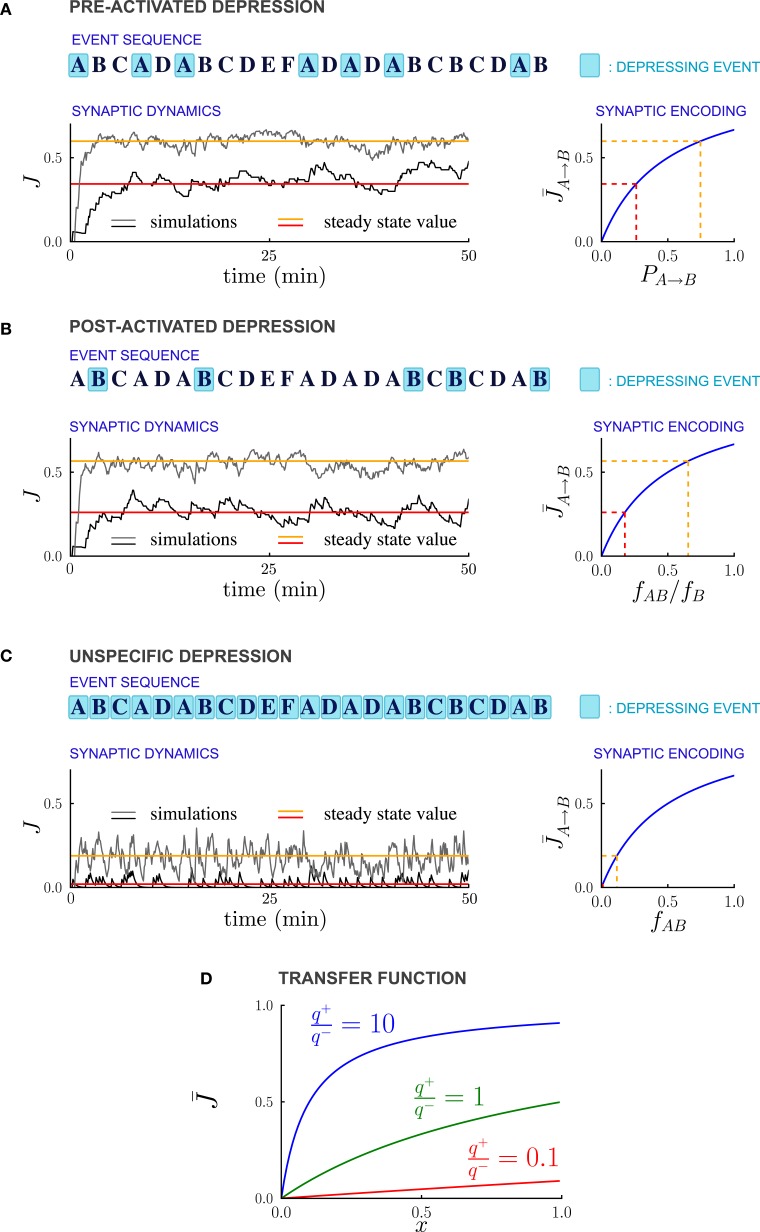
**Synaptic strengths in the steady state encode temporal contiguity. (A–C)** Illustration of the different depression rules, examples of synaptic dynamics, and relationship between steady-state synaptic strengths and temporal statistics of external events, for the three depression rules introduced in the text. **(A)** PRE-Activated depression: the synapses from population *A* to population *B* are depressed each time the pre-synaptic population *A* is activated. The mean synaptic strength in the steady-state encodes the transition probability *P*_*A* → *B*_ from event A to event B. **(B)** POST-Activated depression: the synapses from population *A* to population *B* are depressed each time the post-synaptic population *B* is activated. The steady-state synaptic strength encodes the conditional probability that event A happened at *t* − 1 given that event B happened at *t*. **(C)** Unspecific depression: all the synapses are depressed at every time step. The steady-state synaptic strength encodes the frequency at which the events A and B occur at two consecutive time-steps. In **(A–C)**, the plasticity parameters are *q*_+_ = 0.06 and *q*_−_ = 0.03, and the external events occur at intervals of 600 ms. In the Synaptic Dynamics pannel, the black and the gray traces illustrate the dynamics of two synapses corresponding to two pairs of events that occur with different statistics. The red and orange line indicate the corresponding steady-state values and their and their relationship with event statistics. **(D)** Transfer function *F* that maps from probabilities to average synaptic strength, displayed for of the ratio *q*_+_/*q*_−_ between potentiation and depression.

As in previous studies (Amit and Fusi, 1994; Brunel et al., 1998; Fusi, 2002), we assume that every event potentiates the synapses connecting simultaneously activated neurons. Moreover every event can depress the synapses, and in particular we consider three different forms of depression: (1) *PRE-activated depression*: the synapses connecting a pre-synaptic active to a post-synaptic inactive neuron are depressed; (2) *POST-activated depression*: the synapses connecting a pre-synaptic inactive to a post-synaptic active neuron are depressed; and (3) *Unspecific depression*: all synapses are depressed every time an event occurs (see Figure 2).

Potentiation due to simultaneous activation occurs only between neurons belonging to the same population, and its effects have been studied previously (Amit and Fusi, 1994; Brunel et al., 1998; Fusi, 2002; Fusi and Abbott, 2007). To take into account the temporal aspect of neural dynamics, in our model we include additional plastic modifications induced by sequentially activated neural populations (Sompolinsky and Kanter, 1986; Griniasty et al., 1993). More precisely, we assume that synapses connecting sequentially activated neurons are potentiated: if event A occurs at the current time step, activating neural population *A*, and if the event B occurs at the next time step, activating population *B*, the synapses projecting from *A* to *B* are potentiated. In this study, we assume that the individual events are familiar to the network, so that the synapses connecting neurons belonging to the same population have reached their equilibrium value and remain fixed (Amit and Fusi, 1994; Brunel et al., 1998; Fusi, 2002; Fusi and Abbott, 2007). The pattern of temporal contiguities is, however, novel to the network. We therefore describe only the dynamics of synapses that connect different neural populations. These synapses are potentiated only by the sequential component of synaptic dynamics, that contributes to linking consecutive events.

The rules for modifying the synapses between two different populations are summarized in Figure 1C for the case of PRE-activated depression. The direction of the synaptic modifications are determined by the pre- and post-synaptic activity. We assume that the actual transition to a different synaptic state occurs with a certain probability, which determines the learning rate (large probabilities correspond to fast synapses). Potentiation occurs with probability *q*_+_ and depression with probability *q*_−_. The synaptic modifications depend only on the temporal order of events and not on their timing.

Figure 2 displays examples of synaptic dynamics for a fixed set of transition probabilities for external events, and a given sequence of events. We assume that any two arbitrary neural populations are inter-connected by large number of bistable synapses, and therefore track only the time course of the fraction of potentiated synapses, which determines the overall synaptic strength. Initially, the temporal statistics of the events are unknown, and, we arbitrarily choose all the synapses to be in the depressed state. As the dynamics proceed, the fractions of potentiated synapses between different neural populations stabilize after an initial transient, and then fluctuate around steady state values. How are these steady state values determined by the temporal statistics of events? What is the dynamics of relaxation to this value and what is the source of fluctuations? We analyze these two aspects of the synaptic dynamics in the next two sections.

### 3.1. Steady state: how synaptic distributions encode temporal contiguity

To elucidate the relationship between temporal contiguity and average synaptic strengths at equilibrium, we focus on the dynamics of the population of bistable synapses that project from the neural population encoding event A (denoted as population *A*) to the neural population encoding a distinct event B (denoted as population *B*), A and B being two arbitrary events.

At equilibrium, the fraction of potentiated synapses *J*_*A* → *B*_ fluctuates around a mean value ${\bar{J}}_{A\to B}$ which gives an accurate estimate of the instantaneous synaptic strength if the fluctuations are weak. As the pre- and post-synaptic activity is determined solely by the occurrence of events A and B, the steady state synaptic strength ${\bar{J}}_{A\to B}$ depends only on the frequencies of occurrence of A and B, denoted by *f*_*A*_ and *f*_*B*_, respectively, and on the transition probabilities between A and B denoted by *P*_*A* → *B*_ and *P*_*B* → *A*_. We derived explicit relations between ${\bar{J}}_{A\to B}$ and these temporal statistics of events A and B.

The derivation, described in full mathematical detail in Appendix B, relies on averaging the synaptic dynamics over all possible sequences of events. A crucial aspect of the synaptic dynamics is that potentiation at timestep *t* depends on the sequence of events that occurred at timesteps *t* and *t* − 1 (see Figure 1C). Such sequence-dependent potentiation renders the dynamics sensitive to correlations between consecutive events, which is obviously a desirable property when one needs to encode temporal contiguity. However, at the same time sequence-dependent potentiation introduces correlations in the synaptic dynamics: indeed the probabilities of potentiating the synapses at two consecutive timesteps *t* and *t* + 1 are correlated as they both depend on the event that occurred at timestep *t*.

Although the presence of temporal correlations is a considerable source of complications, we were able to perform a full mathematical analysis of the synaptic dynamics, exposed in detail in Appendix B. In what follows we will analyze the case of slow learning (small potentiation and depression rates *q*_+_ and *q*_−_), but it is important to notice that our approach can deal with the temporal correlations also in the case of fast learning. In the case of slow learning, the steady-state synaptic strength ${\bar{J}}_{A\to B}$ is given by:

(13) $${\bar{J}}_{A\;\to \;B}=F(f_{A\;\to \;B}^{+}/f_{A\;\to \;B}^{-})$$

where *F* is the *synaptic transfer function* defined by

(14) $$F(x)=\frac{\frac{q_{+}}{q_{-}}x}{1+\frac{q_{+}}{q_{-}}x},$$

while *f*^+^_*A* → *B*_ and *f*^−^_*A* → *B*_ are the frequencies of occurrence of potentiating and depressing events (or sequences of events). Notice that an identical result would be obtained if the temporal correlations in the synaptic dynamics are neglected. Although it is not surprising that in the limit of slow learning the correlations may have a small effect on the steady-state distribution of the synapses, it was not clear to what extent the assumption of neglecting the correlations would provide a good approximation. Our mathematical analysis shows in a rigorous way that the correlations can indeed be neglected in the slow learning limit (see Methods and Appendix B).

**Figure 3:**
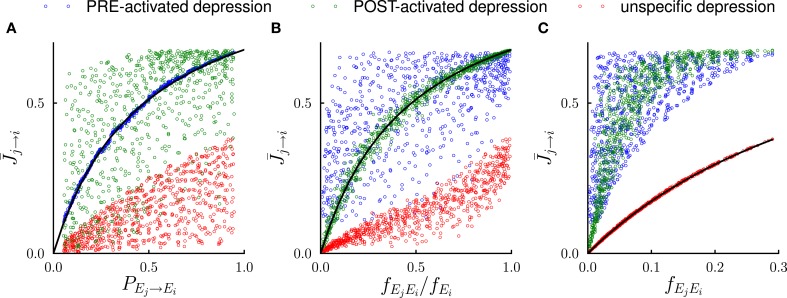
**Systematic comparison between the results of the mathematical analysis and the simulations in the steady state.** In the simulations, the number of external events was fixed to *n* = 12 and 50 Markov transition matrices were generated randomly following the procedure outlined in the Methods. For each transition matrix, the synaptic dynamics were simulated using the three different rules for depression (see Figure 2). For every pair of connected neural populations, the steady state synaptic strength was obtained by averaging over 4000 trials. The three different colors denote the outcomes of the three depression rules. **(A)** Steady state synaptic weights ${\bar{J}}_{j\to i}$ represented as a function of the transition probabilities *P*_*E*_*j*__ → *E*_*i*_. As predicted by the analysis (solid line), for PRE-activated depression the synaptic weights ${\bar{J}}_{j\to i}$ are a function of transition probabilities *P*_*E*_*j*__ → *E*_*i*_, while this is not the case for other depression rules. **(B)** Same data represented as a function of the conditional probability *f*_*E*_*j*_*E*_*i*__/*f*_*E*_*i*__ that event *E*_*j*_ happened at *t* − 1 given that event *E*_*i*_ happened at *t*. For POST-activated depression ${\bar{J}}_{j\to i}$ is a function of *f*_*E*_*j*_*E*_*i*__/*f*_*E*_*i*__ as predicted by the analysis (solid line). **(C)** Same data represented as a function of the frequency *f*_*E*_*j*_*E*_*i*__ at which the events *E*_*j*_ and *E*_*i*_ occur at two consecutive timesteps. For unspecific depression ${\bar{J}}_{j\to i}$ is a function of *f*_*E*_*j*_*E*_*i*__ as predicted by the analysis (solid line).

The steady-state synaptic strengths are nevertheless exquisitely sensitive to the temporal correlations between external events as we now show. In our model, synapses between distinct neural populations are potentiated only due to the sequential activation of the two populations, therefore *f*^+^_*A* → *B*_ = *f*_*AB*_, where *f*_*AB*_ = *P*_*A* → *B*_*f*_*A*_ is the frequency at which the events A and B occur at two consecutive timesteps. The relation between the frequency of depressing events *f*^−^_*A* → *B*_ and the statistic of external events depends on the plasticity rule used for depression (see Figures 2, 3).

For PRE-activated depression, the synapses are depressed whenever the pre-synaptic event A takes place, so that *f*^−^_*A* → *B*_ = *f*_*A*_, and from Equation (13)

(15) $${\bar{J}}_{A\;\to \;B}=F(P_{A\;\to \;B}).$$

The steady state fraction of potentiated synapses ${\bar{J}}_{A\to B}$ is thus a monotonic function of the transition probability *P*_*A* → *B*_, and hence it directly encodes the temporal contiguity of events A and B (see Figures 2A, 3A ).

For POST-activated depression, the synapses are depressed whenever the post-synaptic event *B* takes place, so that *f*^−^_*A* → *B*_ = *f*_*B*_, and from Equation (13)

(16) $${\bar{J}}_{A\;\to \;B}=F(f_{AB}/f_{B}).$$

The quantity *f*_*AB*_/*f*_*B*_ is the conditional probability of event A having happened at time *t* − 1 given that event B happened at time *t*, so that for POST-activated depression, the steady state value ${\bar{J}}_{A\to B}$ of the synaptic weights encodes another aspect of the temporal statistics of events (see Figures 2B, 3B).

Finally for unspecific depression *f*^−^_*A* → *B*_ = 1 as depression occurs at every time step, so that ${\bar{J}}_{A\to B}=F(f_{AB})$ simply represents the frequency at which the events A and B occur at two consecutive timesteps (see Figures 2C, 3C).

The non-linear transfer function that maps probabilities to synaptic strengths is displayed in Figure 2D. This transfer function depends only on the relative balance between depression and potentiation, i.e., the ratio *q*_+_/*q*_−_ where *q*_+_ and *q*_−_ are, respectively the strengths of potentiation and depression. Importantly, for any value of *q*_+_/*q*_−_, the transfer function is monotonic, so that the mapping from transfer probabilities to synaptic weights is one-to-one, implying that synaptic weights encode probabilities. When potentiation is much stronger than the depression (*q*_+_/*q*_−_ > 10), the transfer function saturates, so that the encoding of large transition probabilities is less efficient. However, for *q*_+_/*q*_−_ varying over a wide range the transfer function is close to linear, and the resolution of the encoding is high.

In summary, the synaptic strengths in the steady state represents different aspects of the temporal contiguity of external events depending on the specific form of synaptic long term depression. Importantly, we showed that the synaptic strengths are monotonic functions of the transition probabilities between events, independently of the details of the plasticity rule.

### 3.2. Synaptic fluctuations represent a running estimate of temporal contiguity

We examine in detail the dynamics of the bistable synapses introduced in the previous section. We focus on two intimately related features, the transients leading to the equilibrium and the fluctuations around the average value in the steady state. We discuss here only the case of PRE-activated plasticity, but our analysis is also valid for other depression rules.

**Figure 4:**
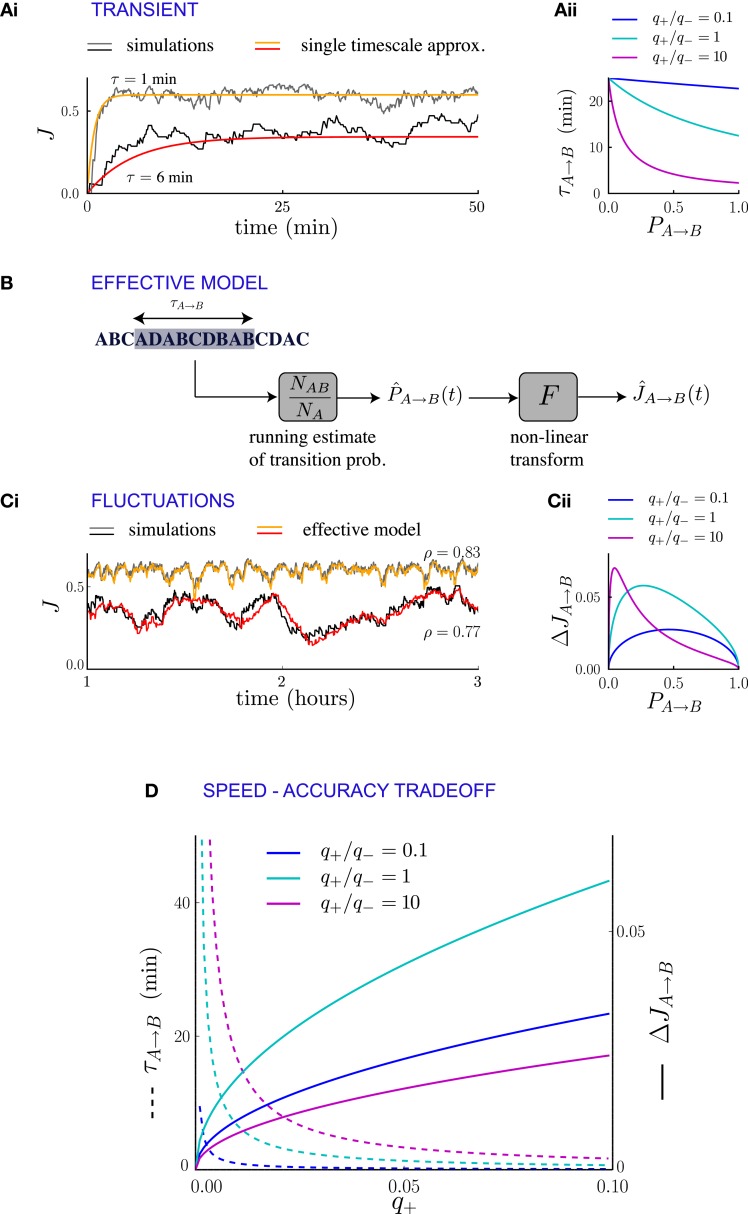
**Synaptic fluctuations represent a running estimate of temporal contiguity. (Ai)** Comparison between numerical simulations (fluctuating lines) and the single timescale approximation (smooth lines), for two synapses corresponding to different transition probabilities. **(Aii)** Transient timescale τ_*A* → *B*_ for the dynamics of synapses projecting from neural population A to neural population B, as function of the transition probability *P*_*A* → *B*_ from event A to event B. The three values of the potentiation-depression ratio *q*_+_/*q*_−_ are obtained by varying *q*_+_ (*q*_+_ = 0.002, 0.02 and 0.2) while *q*_−_ = 0.02 is held fixed. **(B)** Illustration of the effective model for the synaptic dynamics. **(Ci)** Comparison between numerical simulations (gray scale) and the predictions of the effective model (colored lines) of synaptic dynamics. The Pearson's correlation coefficient between simulations and predictions is denoted by ρ. In **(Ai, Ci)**, the plasticity parameters are *q*_+_ = 0.06 and *q*_−_ = 0.03, and the external events occur at intervals of 600 ms. **(Cii)** Standard deviation of the synaptic dynamics in the steady state, as function of the transition probability. The three values of the potentiation-depression ratio *q*_+_/*q*_−_ are obtained by varying *q*_−_ (*q*_−_ = 0.5, 0.05 and 0.05) while *q*_−_ = 0.05 is held fixed. **(D)** Illustration of the speed-accuracy trade-off: the transient timescale τ_*A* → *B*_ is inversely proportional to the potentiation probability *q*_+_, while the amplitude of fluctuations increases as $\sqrt{q_{+}}$. This figure was obtained for *P*_*A* → *B*_ = 0.5. Note that for fixed *P*_*A* → *B*_ = 0.5 and *q*_+_ the fluctuation amplitude is not a monotonic function of *q*_+_/*q*_−_ (see panel **Cii**).

If the statistics of external events change suddenly, the new temporal pattern of events modifies the synaptic dynamics and drives them to a new equilibrium state. The transient of the relaxation dynamics is approximately dominated by a single exponential (Figures 4Ai, 5A), the timescale of which can be computed in terms of transition probabilities and plasticity parameters. If the synaptic dynamics is slow (i.e., if the synaptic transition probabilities *q*_+_ and *q*_−_ are small), this timescale affords a particularly simple mathematical expression. For a population of synapses projecting from neural population *A* to neural population *B*, the timescale of the transient can be directly determined from the averaged synaptic dynamics (see Methods and Appendix B) and reads

(17) $$\tau_{A\;\to \;B}=\frac{{\bar{J}}_{A\;\to \;B}}{q_{+}f_{AB}}$$

where ${\bar{J}}_{A\to B}$ is the average synaptic strength at the new equilibrium, *q*_+_ is the rate of potentiation and *f*_*AB*_ = *f*_*A*_ *P*_*A* → *B*_ is the frequency of occurrence of the sequence AB in the new statistics of external events. This expression is independent of the plasticity rule used for depression, but the value of τ_*A* → *B*_ depends on the depression rule and depression strength *q*_−_ implicitly through ${\bar{J}}_{A\to B}$ (Figure 4Aii). For events occurring at a rate of one per second and potentiation/depression probabilities *q* = 0.02 per event, the transient is of the orders of tens of minutes.

**Figure 5:**
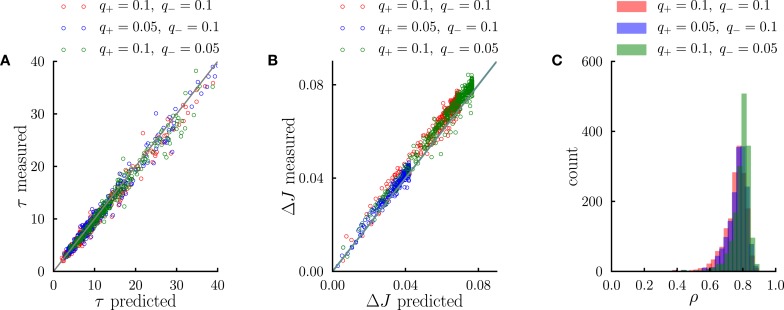
**Systematic comparison between the simulations results and the predictions of the effective model. (A)** Comparison between predicted transient time scales (Equation 5) and time scales obtained from single-exponential fits to transients in numerical simulations. **(B)** Comparison between the standard deviation of synaptic dynamics in the steady-state predicted from the effective model (Equation 11) and the measurements in numerical simulations. **(C)** Distribution of correlation coefficients between synaptic timecourses obtained from simulations and timecourses obtained from the effective model. In the simulations, the number of external events was fixed to *n* = 12 and 10 Markov transition matrices were generated randomly following the procedure outlined in the Methods. For each transition matrix, the synaptic dynamics were simulated using the PRE-activated rule for synaptic depression. In **(A,B)**, each point corresponds to a synaptic population connecting a pair of neural populations. The three different colors denote three different sets of potentiation and depression rates *q*_+_ and *q*_−_.

The transient timescale τ_*A* → *B*_ reflects the time required to collect information about the new statistics of external events. The equilibrium strength of the synapses from *A* to *B* will depend on the transition probability from A to B, which is estimated by accumulating evidence from the observed sequence of events. The mathematical expression for the timescale of the transient is consistent with this interpretation: τ_*A* → *B*_ is inversely proportional to the learning rate *q*_+_, that corresponds to the amount by which the total synaptic strength changes each time the sub-sequence AB is observed. Moreover, τ_*A* → *B*_ is inversely proportional to the frequency of the sub-sequence AB, simply because the synapses are potentiated only when AB occurs. If the relevant sub-sequence is less frequent, it takes longer to accumulate relevant evidence. The fact that τ_*A* → *B*_ is proportional to the asymptotic value ${\bar{J}}_{A\to B}$ of the synaptic strength (which itself is an increasing function of the transition probability from A to B) is less expected, and indicates that the synaptic dynamics do not act as a simple leaky-integrator with a fixed time-constant, but that instead the time constant adapts to the quantity being integrated.

We have interpreted the timescale of the transient as the timescale over which the synapses integrate information from the ongoing dynamics of external events. If this interpretation is correct, it should hold not only during the transient, but also in the steady state, so that the fluctuations of synaptic strengths in the steady state should correspond to the fluctuation of evidence about the transition probability from A to B in a sliding window of extent given by τ_*A* → *B*_. To test this conjecture, we constructed an effective model of synaptic dynamics (Figure 4B), in which the transition probability from event A to event B is estimated in a sliding window of extent τ_*A* → *B*_, simply by computing the ratio between the number of occurrences of the sequence AB divided by the number of occurrences of event A. That estimate of the transition probability is then transformed into a synaptic weight by applying the transfer function described in the previous section (see Figure 2D). The comparison between the synaptic dynamics in the full model and the effective model is displayed in Figure 4C. The correlation coefficients between the two time series range between 0.7 and 0.9 depending on the details of the parameters (see Figure 5C). The transient timescale τ_*A* → *B*_ represents the longest timescale in the dynamics; our effective model mainly reproduces the fluctuations at long timescales, but misses fluctuations at shorter timescales.

The effective model clearly shows that the synaptic dynamics compute the transition probability between events in an ongoing fashion. This model also allows us to compute in a simple manner the magnitude of fluctuations in the steady state as a function of the statistics of external events and synaptic parameters. The corresponding formula is given in Methods, Equation (6), and the result matches very well numerical simulations (see Figure 5B). The standard deviation Δ*J*_*A* → *B*_ of the synaptic strength is inversely proportional to the square root of the integration time scale, as could be expected, but it also depends explicitly on the transition probability, and the ratio between potentiation and depression (Figure 4Cii).

In summary, we have shown the transients and the fluctuations in the synaptic dynamics are closely related due to the fact that synaptic dynamics perform an ongoing computation of temporal contiguity, by accumulating evidence in a sliding window of finite extent. This observation reveals a fundamental speed-accuracy trade-off in the synaptic encoding of temporal contiguity: when the dynamics of external events change, it is important to compute as fast as possible the new transition probabilities, so that the transients remain short. Hence, it seems desirable to have large learning rates (i.e., large probabilities for synaptic potentiation and depression), as the transient timescale is inversely proportional to them (Figure 4). Short transients, however, imply strong fluctuations in the steady state, and thus unreliable estimates, as the synaptic strengths become more sensitive to the fluctuations in the statistics of external events.

### 3.3. Multi-state synapses with hard and soft synaptic bounds

In the previous sections we analyzed how bistable synapses can encode temporal contiguity. In this section we extend that analysis to specific models of multi-state synapses. We first consider a synapse that has to traverse *m* states before reaching its bounds [as in Fusi and Abbott, (2007), see also Methods]. The transition probabilities between synaptic states are independent from the distance to the bounds (hard bounds—see below for the soft bound case). As the number of synaptic states *m* increases, we observe three effects: (1) the synaptic weights become sigmoidal functions of the transition probabilities and these sigmoids become progressively steeper, making it possible to store only a rough estimate of the probabilities (basically only the information about whether the probability is above or below a threshold); (2) the time it takes to reach equilibrium and generate an estimate of the probabilities increases linearly with the distance between the boundaries; and (3) the estimate of the probabilities becomes progressively more “reliable” in the sense that the fluctuations around the estimated value become smaller, as in the case of bistable synapses with smaller learning rates analyzed in the previous section.

**Figure 6:**
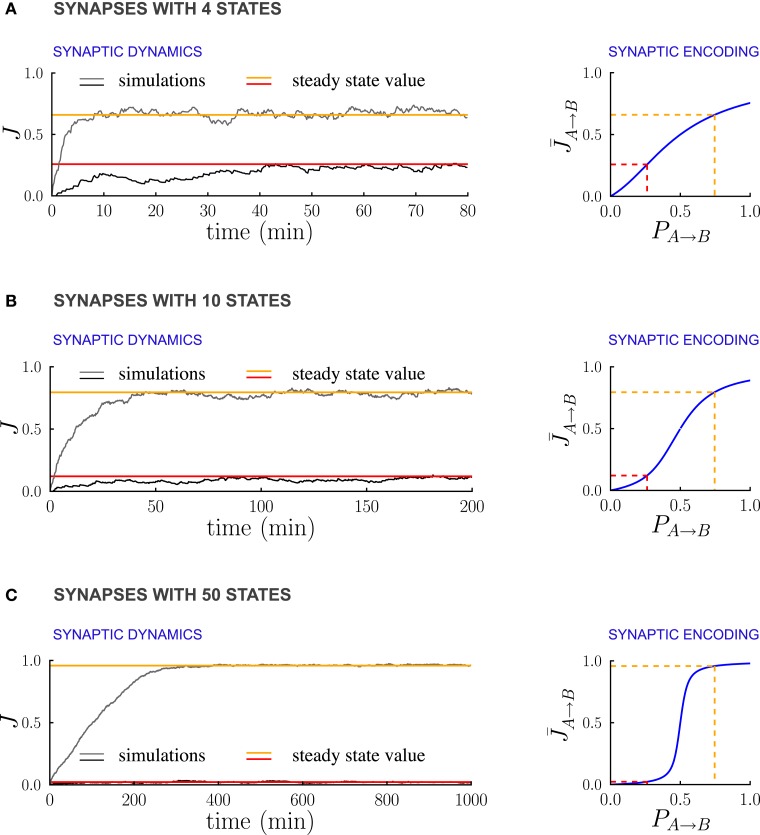
**Multi-state synapses with hard bounds encoding temporal contiguity. (A–C)** Examples of synaptic dynamics (left), and relationship between between steady-state synaptic strengths and transition probabilities (right) for synapses with an increasing number of states: **(A)** 4 states; **(B)** 10 states; **(C)** 50 states. As the number of synaptic states increases, the effective learning rate decreases, the fluctuations around the estimates decrease estimates decrease (see the black traces) and the transfer function [given in Methods, Equation (5)] that relates synaptic weights to transition probabilities becomes an increasingly steep sigmoid centered at *q*_−_/*q*_+_ (*q*_+_ and *q*_−_ being the potentiation and depression rates). In the limit of a large number of synaptic states, the steady state synaptic weights are therefore essentially bistable and encode only whether the transition probabilities are smaller or larger than *q*_+_/*q*_−_. In the Synaptic Dynamics panel, the black and the gray traces illustrate the dynamics of two synapses corresponding to two probabilities. The red and orange line indicate the corresponding steady-state values and their relationship with event statistics. Results are shown for the PRE-activated depression rule, the plasticity parameters are *q*_+_ = 0.06 and *q*_−_ = 0.03, and the external events occur at intervals of 600 ms. Note that for soft-bounds the results would be similar to the bistable case (see the text).

Figure 6 illustrates the dependence of the synaptic dynamics on the number of synaptic states *m*, in the case of PRE-activated depression. For a small number of synaptic states (Figure 6A, *m* = 4), the synaptic dynamics are almost identical to the ones of the bistable case: an equilibrium state is reached quickly, and the average synaptic strengths are approximately a linear function of the transition probabilities (Figure 6A left). As *m* grows, the time needed to reach equilibrium increases, and the synaptic strengths become an increasingly steep sigmoidal function of the transition probabilities (see Figures 6B,C). For synapses with *m* = 50 states (Figure 6C), the synaptic dynamics take a long time to reach equilibrium, and the synaptic strengths eventually encode only whether the transition probability is larger or smaller than the ratio *q*_+_/*q*_−_ between potentiation and depression rates. However, the black traces in (Figure 6) show that the fluctuations of the estimate of the probability decrease progressively as the number of states increases. This is due to the fact that the effective learning rate decreases as the number of states increases and then the probabilities are estimated on a longer time window (see the previous section for a more quantitative analysis of the effects of the learning rate on the accuracy of the estimate). Depending on the task to be performed and on the statistics of the environment, there is an optimal number of synaptic states.

The case of soft boundaries, in which the transition probabilities decrease progressively to zero as the synapse approaches the boundaries [see Fusi and Abbott, (2007) for more details], is qualitatively similar to the case of bistable synapses. The relationship between the synaptic weights in the steady state and the transition probabilities is the same as in the case of a population of bistable synapses. Indeed the dynamic equations for the expected fraction of potentiated synapses are the same as the dynamic equations for the soft boundary synapses. The time needed to reach equilibrium, however, increases linearly with the number of synaptic state, and the fluctuations decrease, in the same manner as in the case of hard boundaries.

### 3.4. Implications for neural dynamics

So far the only assumption about the structure of the network was that it consisted of a number of distinct populations of excitatory neurons, each selective to an external event. We now consider this network after learning has taken place, so that the synaptic weights between the different neural populations are obtained by applying the synaptic transfer function (in Figure 2D) to the transition probabilities between the corresponding external events. We will show that temporal contiguity encoded in the distribution of the synaptic weights, can easily be read out and exploited by a neural circuit. In particular we will show that synaptic encoding of temporal contiguity allows a simple, biologically plausible neural network to perform prospective coding in the sense that when the network is stimulated by the occurrence of an event, it responds by activating the population of neurons that correspond to the event which most often follow A. This basic phenomenology is similar to neural activity observed in the infero-temporal and prefrontal cortex of primates performing a pair-associate task (Sakai and Miyashita, 1991; Rainer et al., 1999) and in the amygdala and orbito-frontal cortex of primates performing a trace-conditioning task (Paton et al., 2006). Notice that this is only one example of possible neural readout of the information stored in the synapses. Other neural circuits may readout the transition probabilities in different ways and they may be able to represent also the information about the less likely transitions.

**Figure 7:**
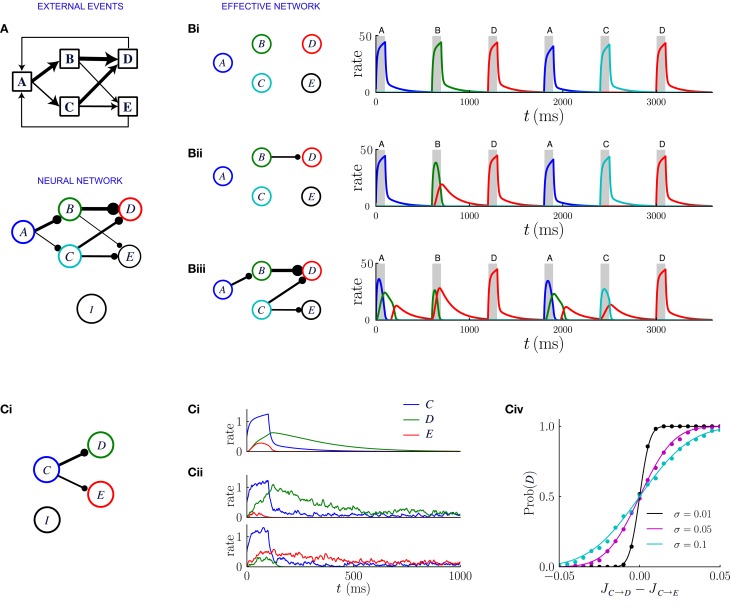
**Network dynamics after learning, for a sequential scheme of external events. (A)** Scheme of external events (top), and the neural network after learning (bottom). The width of the connections represents the magnitude of transition probabilities and corresponding excitatory weights. The inhibitory populations receives uniform synaptic inputs from all excitatory populations, and inhibits all of them equally. **(B)** Dynamics of the network for three different values of the inhibitory strength *g*_*I*_, decreasing from **(Bi)** to **(Biii)**. The effective network (left) represents the excitatory connections that are stronger than the inhibition. The trajectories on the right represent the network dynamics in response to a sequence of external events. **(C)** Noise in the activity leads to probabilistic predictions. **(Ci)** Effective connectivity of a sub-network in which population *C* excites both populations *D* and *E*, with *J*_*C* → *D*_ > *J*_*C* → *E*_, both connections being stronger than the inhibition. **(Cii, Ciii)** Examples of winner take all dynamics after the occurrence of event C. **(Cii)** In absence of noise, the population *D* receiving the strongest synaptic input always wins and inhibits population *E*. **(Ciii)** In presence of noise either *D* or *E* can win, so that the network predicts probabilistically that either event D or E can follow event C. **(Civ)** Probability that population *D* wins as function of the difference between input synaptic weights, for three different levels of noise. The dots represent numerical simulations, and the continuous lines are sigmoidal fits to the data.

The model we consider is a firing rate model which is essentially a simplified version of a biologically realistic network of spiking neurons (Brunel and Wang, 2001; Wang, 2002; Wong and Wang, 2006). The scheme of the network is illustrated in Figure 7A: each excitatory population is activated by only one event, all excitatory populations are connected among themselves with bistable synapses and to a population of inhibitory neurons, which in turn projects to all the excitatory populations. The inhibitory population responds linearly to the total excitatory input and it is not selective to external events, but balances excitatory activity and stabilizes the network.

The dynamical behavior greatly depends on the strength *g*_*I*_ of inhibitory synapses. When an excitatory population *A* is activated, the post-synaptic populations are excited by direct synapses and simultaneously inhibited by feed-forward inhibition. The net effect on the post-synaptic population B will be either excitatory or inhibitory depending on whether the synaptic weight *J*_*A* → *B*_ is greater or weaker than the strength of the inhibition *g*_*I*_. The strength of inhibition therefore determines an effective connectivity matrix between the excitatory populations.

Figure 7 illustrates the network dynamics corresponding to an asymmetric scheme of external events similar to the probabilistic trace-conditioning task described in Figure 1A. The dynamics are described for three different strengths of inhibitory synapses. The synaptic matrix determined by the temporal contiguity is highly asymmetric and essentially feed-forward (Figure 7A). If inhibition is strong (Figure 7Bi), all the excitatory synaptic connections are weaker than inhibition, which basically prevents effective excitatory interactions: when an external event occurs, only the corresponding population is activated, as was the case prior to learning.

For weaker inhibition (Figure 7Bii) some of the excitatory synapses are stronger than the inhibition and the different populations start to interact. The strongest synapses are those that correspond to the largest transition probabilities between events: in the illustration of Figure 7, event A is very often followed by event C, so that the synaptic weight *J*_*A* → *C*_ is larger than the inhibitory strength *g*_*I*_. When event A occurs after learning, the corresponding population *A* is transiently activated, which in turn induces a transient in population *C*: after observing the event A, the network predicts that event C is likely to occur next. The amplitude of the transient activation is moreover proportional to *J*_*A* → *C*_ − *g*_*I*_ and therefore increases with the probability that C will occur after A.

Even weaker inhibition leads to a greater number of excitatory connections stronger than inhibition (Figure 7Biii) and new phenomena emerge in the dynamics of the network. The first is the appearance of sequences of transient activations. If event C is often followed by event E, then when event A occurs, the populations C and E will be sequentially activated, predicting the most likely sequence of events. Each transient is a low-pass version of the previous one, therefore the successive activations in the sequence will be attenuated and elongated in duration, which limits the length of the predictable sequence.

A second possible phenomenon is the appearance of a population that excites more than one post-synaptic population, such as population C in Figure 7Biii. When the event C takes place, because of the presence of the global inhibition, the two post-synaptic populations enter in competition with each other via a winner-take-all mechanism. Eventually one of the two populations takes over and is fully activated. Which population wins depends on the amplitude of the noise in the firing rate dynamics (see Figure 7C). If the noise is weak, the population which receives the strongest excitation always wins, so that the network always predicts the event that is the most likely to follow. If the amplitude of the noise is strong, the post-synaptic population that is activated is chosen stochastically with a probability proportional to the difference between the synaptic weights. Reproducing in the neural dynamics the precise transition probabilities of the corresponding external events would, however, require a tuning of the noise amplitude, or an additional mechanism not included in this simplified framework.

In summary, as a consequence of the synaptic encoding of temporal contiguity, the neural network dynamics provide a predictive model of the external environment, in the case where the scheme of events is sequential. The strength of inhibition in the network determines the timescale over which the predictions are made: the weaker the inhibition, the longer the predicted sequence of following events. The maximal length of sequences that can be predicted is, however, limited by the requirement that inhibition is strong enough to stabilize the excitatory activity in the network. This requirement effectively limits the network to learning the most likely transitions between events rather than the full statistics.

For a scheme of events similar to natural viewing of three-dimensional objects (Figure 1A), the transition probabilities between two events are essentially symmetric, and the synaptic matrix is therefore also symmetric rather than feed-forward. The neural network then consists of modules of populations within which the excitatory interactions are strong, while the interactions between the modules are weak. Networks with similar connectivity have been extensively studied in previous works (Brunel and Wang, 2001), and it has been shown that under suitable conditions the modules become attractors: when an event occurs, the whole corresponding module is activated, possibly in a persistent manner. This mechanism could naturally lead to invariant visual representations: if prior to learning, different neural populations encode the retinal image of an object seen from different angles, due to temporal contiguity in natural vision, these different neural representations merge in a single population selective to all angular projections of the object.

## 4. Discussion

We showed that different statistical aspects of temporal contiguity, and in particular transition probabilities, can easily be encoded in the distribution of the synaptic weights. The ability of synapses to encode temporal contiguity is not an obvious consequence of the dependence of long term synaptic modifications on sequences of pairs of consecutive events. In order to estimate a transition probability, it is necessary to remember multiple instances of pairs of consecutive events. This information must be stored and retained by any neural or synaptic mechanism that is in charge of estimating a probability. Even when this is possible, there is a further complication due to the correlations between successive long term synaptic modifications that are often present in long sequences of events generated by Markov processes. These correlations have never been considered in previous works, as the investigators have focused on individual pairs of consecutive stimuli that were not embedded in longer sequences (see e.g., Soltani and Wang, 2010).

Here we showed that despite these complications, it is possible to encode transition probabilities in the distribution of synaptic weights. We learned that two ingredients are important for synaptic encoding of temporal contiguity: (1) not surprisingly, the long term modifications cannot depend only on individual events, but they must depend at least on pairs of consecutive events and (2) the synaptic weights must be bounded and the synaptic dynamics must be sensitive to these bounds in order to be able to encode the transition probabilities. We now discuss separately the two ingredients. We then comment on some of the simplifying assumptions, present experimental predictions and we finally discuss the general importance of synaptic encoding of transition probabilities.

### 4.1. Sequence-dependent synaptic plasticity

The first necessary ingredient concerns the events triggering long term synaptic changes: not surprisingly, in order to encode the temporal statistics of external events, synaptic modifications must depend on the temporal order of at least two consecutive events. Specifically, in order to encode the transition probability from A to B, it is sufficient to assume that the synapses are modified in one direction (e.g., they are potentiated) when the transition occurs and in the opposite direction (depressed), when it does not occur. This implies that the synapse should be able to detect the temporal sequence A–B, or in terms of pre and post-synaptic activity, the sequential activation of pre-synaptic neurons encoding A and post-synaptic neurons encoding B. One may be tempted to relate the learning rules that we discussed to the observed long term synaptic changes that depend on the temporal order of pre- and post-synaptic stimulations (Levy and Steward, 1983), or on the relative timing of individual pre- and post-synaptic spikes, a property known as spike-time-dependent plasticity (STDP) (Markram et al., 1997; Bi and Poo, 1998; Sjöström et al., 2001). However, in these electro-physiological experiments the typical intervals separating consecutive stimulations are of the order of tens of milliseconds. In contrast, in many behavioral experiments, the events that are temporally contiguous can be separated by seconds or even longer intervals, so STDP cannot explain the learning rule that we propose. However, synaptic mechanisms like STDP can be used to encode some form of temporal contiguity between events that are separated in time. One possible mechanism has been proposed in Drew and Abbott (2006), in which the authors realized that the signal about temporal contiguity can be generated by STDP as long as the relative timing of the pre and post-synaptic spikes is preserved over the time interval separating the relevant events. This is the case when the stimuli elicit sustained activity and this activity is modulated in time, e.g., by adaptation. The mechanism proposed in Drew and Abbott (2006) would be compatible with our rule for potentiating the synapses, but not with the rules for depressing the synapses, which would require some other mechanism. Sustained activity observed in delays between sensory stimuli has been observed in many experiments *in vivo* and is assumed to play an important role in bridging the gap between events that are separated by seconds (Yakovlev et al., 1998). Other possibilities include relying on synaptic mechanisms like synaptic tagging (Clopath et al., 2010), or context-sensitive cells that have been found in the CA3 region of the hippocampus (Wallenstein et al., 1998).

In this study, we have used a macroscopic plasticity rule that does not assume a specific mechanism implementing sequence-dependent synaptic plasticity. How our plasticity rule can be derived from a microscopic plasticity rule (Clopath et al., 2010; Graupner and Brunel, 2012) and what kind of mechanism is needed to bridge time-scales remains to be studied.

### 4.2. Synaptic boundaries and the importance of forgetfulness

The second important ingredient is the existence of an equilibrium distribution for the synaptic weights: the synaptic dynamics should guarantee that the distribution of the synaptic weights converges to a steady-state when the statistics of external events do not change in time. In the case of synapses that are restricted to vary in a limited range (bounded synapses), there is a unique equilibrium distribution for a given statistics of long term modifications, which in turn reflects the statistics of the events to be encoded (Fusi, 2002). In the case considered here, the temporal statistics of the events are encoded in the mean of the equilibrium distribution, but they could alternatively be encoded in the variance or other properties of the distribution.

The importance of the equilibrium distribution is related to the fact that it makes the synapses forgetful, as they always converge to the same distribution, independently from the initial conditions. Basically, forgetful synapses estimate probabilities/transition probabilities by counting events/sequences of events in a sliding temporal window. Events which fall outside of this window are forgotten. Non-forgetful synapses would count all events from their birth. This is the case of synapses which are unbounded and that sum linearly all the induced modifications [similarly to the Hopfield model (Hopfield, 1982)]. In this situation, the synaptic dynamics perform a (biased) random walk, so that the mean and standard deviation of synaptic weights increase over time, and hence there is no equilibrium distribution. These models not only are unrealistic because physical synapses are always bounded, but they are also inefficient at encoding the statistics of the events, because the distribution of synaptic weights keeps changing even when the statistics of the events are stationary.

Interestingly, the boundedness and the consequent existence of an equilibrium distribution is usually highly disruptive for memory performance (see e.g., Fusi, 2002). In contrast, for the encoding of temporal contiguity between events, forgetfulness is a key property. The main difference between the two problems is that in many standard memory benchmarks memories are stored in one shot and then overwritten by other uncorrelated memories. In the case of transition probability estimation, “memories” are repeatedly rewritten, as they are represented by the statistics of the environment, which is continuously sampled by “observing” the occurrence of the events, for as long as the environment does not change.

### 4.3. Speed-accuracy trade-off

As the temporal statistics of the events are estimated by counting events in a sliding temporal window, it is not surprising that our analysis revealed a speed-accuracy trade-off in the encoding of transition probabilities. Indeed, the longer the window (i.e., the smaller the learning rates), the more accurate the estimate. However, for longer temporal windows the convergence to the estimate becomes also slower and this is a limitation when the statistics of events changes. The speed-accuracy trade-off can probably be relaxed in more complex synapses which operate on multiple timescales. Indeed, the fast components of the synaptic dynamics could be utilized to rapidly estimate probabilities/transition probabilities, and the slow components would be devoted to increasing the accuracy of the estimate as the statistics accumulates. Ultimately, the optimal value of temporal window is determined by the rate at which temporal statistics themselves change and a model with multiple timescales might be able to function at this optimal timescale.

In the future, we plan to study synaptic models that operate on multiple timescales, and we believe that models similar to the cascade model (Fusi et al., 2005) can greatly improve the performance. Indeed, the cascade of biochemical process that underlie memory consolidation at the synaptic level could provide us with a wide spectrum of mechanisms operating on different timescales. Notice that our analysis already applies to heterogeneous populations of bistable synapses characterized by different timescales. These heterogeneous populations of simple synapses would already provide the neural circuit with a compromise between fast and slow estimates, although they cannot be as efficient as more complex cascade synapses. The introduction of independent neural systems that can detect changes in the statistics of the events (Yu and Dayan, 2005), could probably further improve the performance and relax even more the constraints about speed and accuracy. This system would probably affect the statistics of the synapses by changing their plasticity through neuromodulation.

### 4.4. Neural representations of the events

In our model, we assumed that the neural network consisted of distinct neuronal populations selective to different external events. Neurons selective to complex stimuli have been commonly found in the ventral and frontal parts of the cortex. In general, a given neuron is selective to more than one stimulus, implying that the different neural populations display some overlap (Yamane et al., 2006). How large these overlaps are is matter of ongoing studies, and probably depends on the nature of the stimuli and the task being performed. In this study, we have analyzed the case in which the neural populations representing different events do not overlap. Our analysis is still a good approximation in the limit of random sparse representations, i.e., when overlap between neural populations is small. For more distributed representations, we expect that overlaps will act as a source of additional noise in the synaptic encoding of temporal contiguity. Our results nevertheless still hold for all the synapses connecting the non-overlapping parts of the neural populations.

### 4.5. Experimental predictions

The work by Levy and Steward, (1983) indicates that long term modifications depend on the temporal order of pre and post-synaptic stimulation. According to our theory and under the numerous simplifying assumptions on the synaptic dynamics, this should be sufficient to encode the temporal statistics of events represented by the activation of the pre and post-synaptic neurons. Our prediction is that the average synaptic weight of a population of synapses should be a monotonic function of one or more aspects of the temporal statistics of the events. This prediction can be tested by (1) generating a sequence of events with a Markov process, (2) computing the corresponding temporal sequence of activation of a representative pair of neurons (e.g., neurons connecting two events that are temporally contiguous in a sufficient number of cases), and (3) stimulating repeatedly the pre and post-synaptic neuron to induce a series of long term synaptic modifications, as in the experiments described in O'Connor et al., (2005), in which the authors used only deterministic sequences. If the procedure is repeated for a sufficient number of synapses, then it is possible to estimate the average value of a population of synaptic weights that is exposed to the same statistics of events. According to our theory, it should be possible to manipulate the mean synaptic weight by changing the temporal statistics of events. This type of experiments not only can confirm our prediction, but they can also give important indications on the way synapses are modified every time the pre- and post-synaptic neurons are activated, and on the most efficient experimental protocols to encode different aspects of the statistics of temporal contiguity.

### 4.6. The importance of synaptic encoding of transition probabilities

Uncertainty is an important component of life, and we have to deal with it in many situations. In some of them, uncertainty derives from the fact that our environment is only partially observable and we need to combine multiple pieces of evidence and our prior knowledge to reach a conclusion about the identity of sensory stimulus. In some other, the environment is inherently stochastic or it is difficult to predict with certainty (e.g., the weather). In all these situations it is useful to represent in the brain the distribution of probabilities of different outcomes, and, in the case of sensory stimuli that vary in time, to estimate the parameters that characterize the temporal statistics of the stimuli and of the effects of our actions on the environment. A number of recent studies have investigated how the brain performs probabilistic inference to perform optimally in uncertain environments (Körding and Wolpert, 2004; Ma et al., 2006; Berkes et al., 2011). In all these cases it is necessary to estimate and encode the prior probability that a particular sensory stimulus is observed. In some of these studies, the authors showed that it is possible to perform Bayesian statistical inference by combining the information about the probability distribution of the relevant variables. If these distributions are encoded in the variability across neurons, then it is possible to implement in a simple neuronal circuit a process that leads to optimal decision making (Ma et al., 2006). In Ma et al., (2006), the authors assumed that the distributions are already encoded in the pattern of neuronal activities but they did not describe the process of probability encoding. In Soltani and Wang, (2010) the authors show a simple case in which the posterior probability that a choice alternative is correct given a particular sensory cue is encoded in the average synaptic strength. Although the analyzed case is very simple, their work is interesting because it shows that it is possible to encode probabilities in a realistic neural circuit (and specifically in the synapses) and then perform optimal decision making.

In our work we showed that these type of posterior probabilities are naturally encoded by synapses also when the correlations between long sequences of consecutive events are taken into consideration [in Soltani and Wang, (2010) the authors considered only the transition probability from the presentation of a cue to the choice of a rewarded action and they did not consider the correlation between two successive synaptic modifications as the trials in the task they analyzed are uncorrelated]. We believe that this an important step toward the understanding of the biological substrate of the successor representation in reinforcement learning (Dayan, 1993; Gershman et al., 2012), and more generally for Markov Decision Processes (Bellman, 1957), which play an important role for decision making in complex tasks that are based on multiple choices (Sutton and Barto, 1998). In these processes, the optimal strategy can be found by trial and error by combining the information about the transition probability between two states of the environment induced by the execution of a particular action, and the immediate reward delivered to the agent. We believe it is possible to encode these conditional transition probabilities by using conjunctive representations of the type described in Rigotti et al. (2010), in which neurons would naturally respond to a particular state only if a certain action is executed. These neurons can be easily obtained with random connectivity. Showing that it is possible to construct a realistic neural circuit that encodes the relevant quantities for learning Markov Decision Processes will be the next important step.

In our analysis we studied how temporal contiguity is encoded in populations of synaptic weights. In real world problems, in many situations one may need to consider higher order temporal correlations, or in other words, the problem of encoding non-Markov processes where the probability of an event depends on a certain number of previous events. These problems are known to be difficult and there are only a few models of neural circuit that address these issues (see e.g., Jensen and Lisman, 1996).

### Conflict of interest statement

The authors declare that the research was conducted in the absence of any commercial or financial relationships that could be construed as a potential conflict of interest.

## Appendix

### A. Mathematical analysis—bistable synapses

Here we present the full mathematical analysis of dynamics of bistable synapses. The method we used is essentially a generalization of the approach introduced in Brunel et al., (1998), where synaptic dynamics were studied in the context of the learning of events occurring in uncorrelated sequences. In contrast, here we focus on learning the temporal correlations between events. We first study the dynamics in the case of arbitrary potentiation and depression probabilities *q*_+_ and *q*_−_. In a second step, we examine the slow learning limit of small *q*_+_ and *q*_−_.

We consider a population of bistable synapses connecting the pre-synaptic neural population *j* (selective to the event *E*_*j*_) to a post-synaptic neural population *i* (selective to the event *E*_*i*_), with *i* ≠ *j*. The analysis is valid for any arbitrary *i* an *j*, we therefore drop these indices and simply refer to neural populations (resp. events) *i* and *j* as the post-synaptic and the pre-synaptic neural population (resp. external event). From the point of view of the population of synapses, only three different external events can occur: (1) the pre-synaptic event; (2) the post-synaptic event; and (3) an event that is neither the pre-synaptic nor the post-synaptic event (all such events are equivalent for the considered synapses). In the following we therefore work with a reduced set of events in which all the events other than the pre and post-synaptic one are identified.

To track which event took place at time *t*, we introduce a variable *h*_*t*_ which takes on values *h*_*t*_ = 1, 2, 3, respectively for the three types of events out of the reduced set. The statistics of the sequence *h*_*t*_ are fully determined by the transition probabilities between the full set of events. While the original stochastic process on the full set of events had the Markov property, this is not necessarily the case for the stochastic process *h*(*t*) on the reduced set of events. We nevertheless use a mean-field approach and approximate *h*(*t*) by a Markov process on the reduced set. The reduced transition matrix Π, where Π_*lm*_ is the probability that *h*_*t*_ = l given that *h*_*t* − 1_ = *m* can be expressed in terms of transition probabilities between events in the full set and reads:

(A1) $$\Pi =\left(\begin{array}{ccc} P_{E_{j}\to E_{j}} & P_{E_{i}\to E_{j}} & \sum_{k\neq i,j}f_{E_{k}}P_{E_{k}\to E_{j}}/\\ & & \sum_{k\neq i,j}f_{E_{k}}\\ P_{E_{j}\to E_{i}} & P_{E_{i}\to E_{i}} & \sum_{k\neq i,j}f_{E_{k}}P_{E_{k}\to E_{i}}/\\ & & \sum_{k\neq i,j}f_{E_{k}}\\ 1-P_{E_{j}\to E_{i}}- & 1-P_{E_{i}\to E_{i}}- & \sum_{k,l\neq i,j}f_{E_{k}}P_{E_{k}\to E_{l}}/\\ P_{E_{j}\to E_{j}} & P_{E_{i}\to E_{j}} & \sum_{k\neq i,j}f_{E_{k}} \end{array}\right).$$

The steady-state distribution π^(0)^ of the above Markov matrix is simply given by the steady state frequencies of events *E*_*j*_ and *E*_*i*_ in the full stochastic process: π^(0)^ = (*f*_*E*_*j*__, *f*_*E*_*i*__, 1 − *f*_*E*_*j*__ − *f*_*E*_*i*__). Similarly, the second-order statistics for the pre-synaptic and post-synaptic event are preserved in the reduced description. This is, however, not necessarily the case for higher order statistics.

The individual synapses are bistable, i.e., they possess two discrete levels indexed by *k* = 1, 2, the level *k* = 1 being the depressed level, and *k* = 2 being the potentiated level. In response to external events, the synapses hop stochastically between the potentiated and the depressed level. To describe these stochastic dynamics, we focus on the probability of the occupation of different levels, i.e., the vector ρ in which the *k*th component ρ_*k*_ represents the probability that a synapse is found in the *k*th potentiation level. The dynamics of the distribution ρ is driven by the plasticity occurring due to external events, and can be formulated as an inhomogeneous Markov process (Fusi, 2002):

(A2) ρ(t+1)=M(t)ρ(t).

Here ***M***(*t*) is a Markov transition matrix that specifies the plasticity at time *t*. By hypothesis the plasticity at time *t* depends only on the events that occurred at times *t* and *t* − 1, so that ***M***(*t*) is a function of *h*_*t*_ and *h*_*t* − 1_.

The plasticity rules are fully specified by ascribing a potentiation and a depression probability to each combination of *h*_*t*_ and *h*_*t* − 1_, which amounts to specifying two matrices ***Q***^+^ and ***Q***^−^ of dimensions three-by-three. The entry ***Q***^+^_*lm*_ (resp. ***Q***^−^_*lm*_) represents the potentiation (resp. depression) probability following the combination of events *h*(*t*) = *l* and *h*_*t* − 1_ = *m* with 1 ≤ *l* ≤ 3 and 1 ≤ *m* ≤ 3. Once the matrices ***Q***^+^ and ***Q***^−^ are given, the plasticity matrix ***M*** can be expressed as  where  is the unit 2 × 2 matrix, and $\hat{P}$ and $\hat{D}$ are the potentiation and depression matrices defined by

(A4) $$\hat{P}=\left(\begin{array}{cc} -1 & 0\\ 1 & 0 \end{array}\right)\;\hat{D}=\left(\begin{array}{cc} 0 & 1\\ 0 & -1 \end{array}\right)$$

Iterating Equation (A2), the distribution ρ(*t*) following a sequence of *t* events *h*_1_ … *h*_*t* − 1_ *h*_*t*_ is given by

(A5) $$\rho (t+1)=M(h_{t},h_{t-1})M(h_{t-1},h_{t-2})\ldots M(h_{2},h_{1})\rho_{1}.$$

Our aim is to compute the mean synaptic distribution 〈ρ(*t*)〉 obtained by averaging over all possible sequences of events *h*_1_ … *h*_*t* − 1_ *h*_*t*_. The probability of a given sequence of events *h*_1_ … *h*_*t* − 1_ *h*_*t*_ is given by

(A6) $$\text{Prob}(h_{t}h_{t-1}\ldots h_{1})=\Pi_{h_{t},h_{t-1}}\Pi_{h_{t-1},h_{t-2}}\ldots \Pi_{h_{2},h_{1}}\text{Prob}(h_{1})$$

where the transition matrix Π is defined in Equation (A1), and Prob(*h*_1_) is an initial distribution of events, which, for simplicity, we take to be the stationary distribution π^(0)^, i.e., the right eigenvector with unit eigenvalue of the transition matrix Π. Multiplying Equation (A5) by (A6), and summing over all possible sequences, we get

(A7) $$\begin{array}{l} \langle \rho (t+1)\rangle =\sum_{h_{t},h_{t-1},\ldots h_{1}}[M(h_{t},h_{t-1})M(h_{t-1},h_{t-2})\ldots \\ \;M(h_{2},h_{1})\rho_{1}\Pi_{h_{t},h_{t-1}}\Pi_{h_{t-1},h_{t-2}}\ldots \Pi_{h_{2},h_{1}}\pi^{\left(0\right)}] \end{array}$$

where 1 ≤ *h*_*s*_ ≤ 3 for *s* = 1 … *t*. Note that for fixed *h*_*s*_ and *h*_*s* − 1_, ***M***(*h*_*s*_, *h*_*s* − 1_) is a matrix while Π_*h*_*s*_, *h*_*s* − 1__ is a scalar, so that 〈ρ(*t*)〉 can be written as

(A8) $$\langle \rho (t+1)\rangle =S(t)\rho_{1},$$

with ***S***(*t*) a two-by-two matrix dependent on time.

We will show that *S*(*t*) can be written as  where *B*(*t*) and *C*(*t*) are scalar coefficients that we will compute. For *t* large, ***S***(*t*) becomes time independent, i.e., *B*(*t*) → *B*_∞_ and *C*(*t*) → *C*_∞_ with *B*_∞_ + *C*_∞_ = 1, so that ρ(*t*) → (1 − *B*_∞_, *B*_∞_).

We now proceed to demonstrate Equation (A9) and compute *B*(*t*). In contrast to uncorrelated sequences of external events studied in Brunel et al. (1998), Equation (A7) cannot be factorized as each factor ***M***(*h*_*s*_, *h*_*s* − 1_) or Π_*h*_*s*_, *h*_*s* − 1__ depends on the external events on two consecutive time steps. Instead we iteratively sum over *h*_*s*_ for *s* = 1 … *t* and proceed by induction. To this end we introduce

(A10) $$\begin{array}{l} R(h_{s},s)=\sum_{h_{s-1},\ldots h_{1}}[M(h_{s},h_{s-1})M(h_{s-1},h_{s-2})\ldots M(h_{2},h_{1})\\ \;\Pi_{h_{s},h_{s-1}}\Pi_{h_{s-1},h_{s-2}}\ldots \Pi_{h_{2},h_{1}}\pi^{\left(0\right)}] \end{array}$$

where ***R***(*h*, *s*) is a two-by-two matrix that depends on the event *h* (with 1 ≤ *h* ≤ 3) that occurs at the timestep *s*. The matrix ***S*** at time *t* is directly related to the matrix ***R*** by

(A11) $$S(t)=\sum_{h=1}^{3}R(h,t).$$

From Equation (A7), the evolution of the matrix *R* is given by

(A12) $$R(h,s+1)=\sum_{l=1}^{3}M(h,l)\Pi_{hl}R(l,s).$$

We next show by induction that for any event *h* (with 1 ≤ *h* ≤ 3) and any timestep *s* (with 1 ≤ *s* ≤ *t*) the matrix ***R***(*h*, *s*) can be written as  where  is the unit 2 × 2 matrix, $\hat{P}$ and $\hat{D}$ are the potentiation and depression matrices defined in Equation (A4), and α(*h*, *s*), β(*h*, *s*), and γ(*h*, *s*) are scalar coefficients that depend on the event *h* and the timestep *s*.

For *s* = 1, , so that Equation (A13) is clearly valid with α(*h*, 1) = π^(0)^_*h*_ (where π^(0)^ is the steady state of the transition matrix Π), β(*h*, *s*) = γ(*h*, *s*) = 0.

Assuming that Equation (A13) is valid at the timestep *s*, using the evolution equation Equation (A12), and the expression given in Equation (A3) for the plasticity matrix ***M***(*k*, *l*), we find that ***S***(*k*, *s* + 1) is of the form postulated in Equation (A13), the coefficients at the two consecutive time steps being related by:

(A14) $$\alpha (h,s+1)=\sum_{l=1}^{3}\Pi_{hl}\alpha (l,s)$$

(A15) $$\begin{array}{l} \beta (h,s+1)=\sum_{l=1}^{3}[\Pi_{hl}\beta (l,s)+Q_{hl}^{+}\Pi_{hl}\alpha (l,s)\\ \;-\;(Q_{hl}^{+}+Q_{hl}^{-})\Pi_{hl}\beta (l,s)] \end{array}$$

(A16) $$\begin{array}{l} \gamma (h,s+1)=\sum_{l=1}^{3}[\Pi_{hl}\gamma (l,s)+Q_{hl}^{-}\Pi_{hl}\alpha (l,s)\\ \;-\;(Q_{hl}^{+}+Q_{hl}^{-})\Pi_{hl}\gamma (l,s)]. \end{array}$$

The Equations (A14–A16) can be easily solved: as α(*l*, 1) = π^(0)^_*l*_, we have α(*l*, *t*) = π^(0)^_*l*_ for all *t*. Using vector notation β = (β_1_, β_2_, β_3_) and β = (γ_1_, γ_2_, γ_3_), the dynamics of β(*t*) and γ(*t*) are given by

(A17) $$\beta (t+1)=A\beta (t)+Q^{+}\pi^{\left(0\right)}$$

(A18) $$\gamma (t+1)=A\gamma (t)+Q^{-}\pi^{\left(0\right)}$$

where **A** is a time-independent three-by-three matrix defined by

(A19) $$A=\Pi -(Q^{+}+Q^{-})*\Pi$$

and ^*^ denotes the element-by-element product of matrices.

The steady state β_∞_ is simply given by

The dynamics of β(*t*) are determined by the time-independent matrix **A**, which has a zero eigenvalue corresponding to the steady state, and two eigenvalues of norm less than one. The relaxation to the steady state of β(*t*) can therefore be expressed as a sum of two decaying exponentials.

Once the evolution of β(*t*) is known, the dynamics of the synaptic occupation probabilities ρ(*t*) are recovered via Equations (A11) and (A8). The synaptic dynamics are then fully specified in terms of the plasticity matrices ***Q***^+^ and ***Q***^−^, and the matrix Π that depends on the statistics of external events.

While the calculation presented above yields a full description of synaptic dynamics, it does not provide a simple and transparent relation between the synaptic dynamics and the statistics of external events. Here we use perturbation theory to uncover such a relationship in the case of slow plasticity. In that limit, we show that the expressions for mean synaptic strengths and transient timescales are identical to those derived above by neglecting correlations in the synaptic dynamics.

We assume that the synaptic transition probabilities specified by the matrices ***Q***^+^ and ***Q***^−^ are small, so that we write

(A21) $$Q^{+}=\epsilon {\hat{Q}}^{+},Q^{-}=\epsilon {\hat{Q}}^{-}.$$

We next introduce a 9-dimensional vector ***X***(*t*) defined by

(A22) $$X(t)\text{​}=\text{​}(\alpha_{1}(t),\alpha_{2}(t),\alpha_{3}(t),\beta_{1}(t),\beta_{2}(t),\beta_{3}(t),\gamma_{1}(t),\gamma_{2}(t),\gamma_{3}(t))$$

or in block notation

(A23) X(t)=(α(t),β(t),γ(t)).

The evolution of the vector *X*(*t*) is given by Equations (A14–A16), which can be rewritten as a single matrix equation:  with $\hat{\Pi }$ and  two nine-by-nine matrices defined in block form by  where ^*^ denotes the element-by-element product of matrices.

To determine the steady state and dominant timescale in the dynamics of ***X***(*t*), we treat  as a weak perturbation to the matrix $\hat{\Pi }$, and use perturbation theory.

In absence of perturbation, for ϵ = 0, the dynamics of ***X***(*t*) are determined by the eigenvalues and eigenvectors of the matrix $\hat{\Pi }$, which can be easily determined. The transition matrix Π possesses three eigenvalues (1, λ_1_, λ_2_). These eigenvalues are also eigenvalues of $\hat{\Pi }$, but with multiplicity three. The left and right eigenvectors of Π associated with the unit eigenvalue are, respectively the unit vector (1, 1, 1) and the steady-state π^(0)^. Three right eigenvectors of $\hat{\Pi }$ that span the subspace associated with unit eigenvalue are therefore

(A25) $$r_{1}=(\pi^{\left(0\right)},\pi^{\left(0\right)},\pi^{\left(0\right)}),\;r_{2}=(\pi^{\left(0\right)},\pi^{\left(0\right)},0),\;r_{3}=(\pi^{\left(0\right)},0,0).$$

The associated left eigenvectors are

(A26) $$l_{1}=(0,0,1),\;l_{2}=(0,1,-1),\;l_{3}=(1,-1,0).$$

When the perturbation is turned on, for ϵ small, the three-dimensional subspace corresponding spanned by ***r***_1_, ***r***_2_, and ***r***_3_ splits into three distinct eigenspaces. To compute the corresponding eigenvalues and eigenvectors, we restrict the matrix O to the subspace spanned by ***r***_1_, ***r***_2_, and ***r***_3_, and compute the eigenvalues (μ_1_, μ_2_, μ_3_) and eigenvectors of the corresponding matrix  defined by

If the three-dimensional vector (*u*^(*k*)^_1_, *u*^(*k*)^_2_, *u*^(*k*)^)_3_ is a right eigenvector of $\bar{O}$ with eigenvalue μ^(*k*)^, the nine-dimensional vector *u*^(*k*)^_1_***r***_1_ + *u*^(*k*)^_2_***r***_2_ + *u*^(*k*)^_3_***r***_3_ is a right eigenvector of the matrix  associated with the eigenvalue 1 + μ^(*k*)^.

We find that the dominant eigenvalue of the matrix $\bar{O}$ is zero with multiplicity one. The corresponding eigenvector is (1, − 1 + ∑^3^_*k*, *l* = 1_(*Q*^+^_*kl*_)Π_*kl*_π^(0)^_*l*_/∑^3^_*k*, *l* = 1_(*Q*^−^_*kl*_)Π_*kl*_π^(0)^_*l*_, 1), which gives the mean steady state potentiation of the synapse:

(A28) $$\bar{J}=\frac{\sum_{k,l=1}^{3}Q_{kl}^{+}\Pi_{kl}\pi_{l}^{\left(0\right)}}{\sum_{k,l=1}^{3}Q_{kl}^{-}\Pi_{kl}\pi_{l}^{\left(0\right)}+\sum_{k,l=1}^{3}Q_{kl}^{+}\Pi_{kl}\pi_{l}^{\left(0\right)}}.$$

The next eigenvalue of $\bar{O}$ is μ_2_ = − ∑^3^_*k*, *l* = 1_(*Q*^+^_*kl*_ + *Q*^−^_*kl*_)Π_*kl*_π^(0)^_*l*_, so that the dominant timescale in the dynamics is given by

(A29) $$\tau =-\frac{1}{\mu_{2}}$$

(A30) $$=\frac{\bar{J}}{\sum_{k,l=1}^{3}Q_{kl}^{+}\Pi_{kl}\pi_{l}^{\left(0\right)}}$$

The outcome of the perturbation calculation summarized in Equations (A28) and (A30) simplifies when particular learning rules are taken into account. Here we summarize the results for the three learning rules described in the main text. These three learning rules differ by the plasticity rule for depression, but the rule for potentiation is identical in the three and given by

$$Q^{+}=\left(\begin{array}{ccc} 0 & 0 & 0\\ q_{+} & 0 & 0\\ 0 & 0 & 0 \end{array}\right).$$

Independently of the details of the depression rule, the dominant timescale of the dynamics is given by

(A31) $$\tau_{j\to i}=\frac{\bar{J}}{q^{+}f_{E_{j}}P_{E_{j}\to E_{i}}}.$$

The mean synaptic strength $\bar{J}$, however, depends on the depression rule.

*PRE-Activated depression* The plasticity rule for depression is given by

$$Q^{-}=\left(\begin{array}{ccc} q_{-} & q_{-} & q_{-}\\ 0 & 0 & 0\\ 0 & 0 & 0 \end{array}\right).$$

which yields

(A32) $${\bar{J}}_{j\to \text{i}}=\frac{\frac{q_{+}}{q_{-}}P_{E_{j}\to E_{i}}}{1+\frac{q_{+}}{q_{-}}P_{E_{j}\to E_{i}}}$$

*POST-Activated depression* The plasticity rule for depression is given by:

$$Q^{-}=\left(\begin{array}{ccc} 0 & 0 & 0\\ q_{-} & q_{-} & q_{-}\\ 0 & 0 & 0 \end{array}\right).$$

which yields

(A33) $${\bar{J}}_{j\to \text{i}}=\frac{\frac{q_{+}}{q_{-}}P_{E_{j}\to E_{i}}f_{E_{j}}/f_{E_{i}}}{1+\frac{q_{+}}{q_{-}}P_{E_{j}\to E_{i}}f_{E_{j}}/f_{E_{i}}}$$

*Unspecific depression* The plasticity rule for depression is given by:

$$Q^{-}=\left(\begin{array}{ccc} q_{-} & q_{-} & q_{-}\\ q_{-} & q_{-} & q_{-}\\ q_{-} & q_{-} & q_{-} \end{array}\right).$$

which yields

(A34) $${\bar{J}}_{j\to \text{i}}=\frac{\frac{q_{+}}{q_{-}}f_{E_{j}}P_{E_{j}\to E_{i}}}{1+\frac{q_{+}}{q_{-}}f_{E_{j}}P_{E_{j}\to E_{i}}}$$

In summary, for the three plasticity rules described in the main text, the result of the full mathematical analysis in the limit of slow learning is identical to the result obtained by ignoring correlations between *J*(*t*) and ξ^+^(*t*, *t* − 1) as well as ξ^−^(*t*), or equivalently between *M*(*t*) and ρ(*t*) in Equation (A2).

### B. Mathematical analysis—synapses with an arbitrary number of states *m*

The mathematical analysis performed for bistable synapses can be extended to the case where synapses possess *m* > 2 states. The full analysis is too cumbersome to be presented here, but the final result is identical to the bistable case: the correlations between *J*(*t*) and ξ^+^(*t*, *t* − 1) as well as ξ^−^(*t*) are negligible in the limit of slow learning (small *q*_+_ and *q*_−_). Here we adopt this result and proceed to compute the transfer function between transition probabilities and average synaptic weights in the steady state, in the case of PRE-activated depression. This computation is based on the derivation performed in Fusi and Abbott (2007).

For synapses with *m* states, the dynamics are still described by Equations (A2), (A3), but the Markov transition matrix that specifies the dynamics is now *m* × *m*. The potentiation and depression matrices $\hat{P}$ and $\hat{D}$ are also *m* × *m* and read

(B1) $$\hat{P}=\left(\begin{array}{ccccc} -1 & 0 & \ldots & \ldots & 0\\ 1 & -1 & \ddots & & \vdots \\ 0 & 1 & \ddots & \ddots & \vdots \\ \vdots & \ddots & \ddots & \ddots & 0\\ 0 & \ldots & 0 & 1 & -1 \end{array}\right)\hat{D}=\left(\begin{array}{ccccc} -1 & 1 & 0 & \ldots & 0\\ 0 & -1 & 1 & \ddots & \vdots \\ \vdots & \ddots & \ddots & \ddots & 0\\ \vdots & & \ddots & \ddots & 1\\ 0 & \ldots & \ldots & 0 & -1 \end{array}\right)$$

Ignoring correlations between ***M***(*t*) and ρ(*t*) in Equation (A2) and averaging over external events yields the averaged synaptic dynamics

(B2) $$\rho (t+1)=\bar{M}\rho (t).$$

with  where *f*_+_ and *f*_−_ are the frequencies of potentiating and depressing events.

The steady state synaptic weights are determined by the eigenvector of $\bar{M}$ associated with the unit eigenvalue. The matrix $\bar{M}$ is a tridiagonal Toeplitz matrix with perturbed corners, and its eigenvectors can be computed by using the ansatz ρ_*k*_ = ξ*r*^*k*^ where ξ and *r* are real numbers to be determined. The result is that the components of the eigenvector associated with unit eigenvalue read

(B4) $$\rho_{k}=\xi {\left(\frac{q_{+}f_{+}}{q_{-}f_{-}}\right)}^{k}$$

and ξ is a normalization constant determined by the requirement that ∑^*m*^_*k* = 1_ ρ_*k*_ = 1.

The average synaptic weight is then given by

(B5) $$J=\frac{1}{m-1}\sum_{k=1}^{m}\left(k-1\right)\rho_{k}$$

(B6) $$=F\left(\frac{f_{+}}{f_{-}}\right)$$

where the synaptic transfer function *F* reads

(B7) $$F(x)=\frac{1}{m-1}\left(\frac{\frac{q_{+}}{q_{-}}x}{1-\frac{q_{+}}{q_{-}}x}+\frac{m{\left(\frac{q_{+}}{q_{-}}x\right)}^{m}}{{\left(\frac{q_{+}}{q_{-}}x\right)}^{m}-1}\right).$$
